# Radiation Belt Storm Probes Ion Composition Experiment (RBSPICE) Revisited: In-Flight Calibrations, Lessons Learned and Scientific Advances

**DOI:** 10.1007/s11214-023-00991-x

**Published:** 2023-11-28

**Authors:** Matina Gkioulidou, Donald G. Mitchell, Jerry W. Manweiler, Louis J. Lanzerotti, Andrew J. Gerrard, Aleksandr Y. Ukhorskiy, Kunihiro Keika, Christopher G. Mouikis, Lynn M. Kistler

**Affiliations:** 1 Applied Physics Laboratory, Johns Hopkins University, Laurel, MD USA; 2 Fundamental Technologies, LLC, Lawrence, KS USA; 3 Center for Solar-Terrestrial Research, New Jersey Institute of Technology, Newark, NJ USA; 4 Department of Earth and Planetary Science, Graduate School of Science, University of Tokyo, Tokyo, Japan; 5 Space Science Center, University of New Hampshire, Durham, NH USA

**Keywords:** Magnetosphere, Ring current, Time-of-flight, Space weather

## Abstract

The Radiation Belt Storm Probes Ion Composition Experiment (RBSPICE) on both the Van Allen Probes spacecraft is a time-of-flight versus total energy instrument that provided ion composition data over the ring current energy (∼7 keV to ∼1 MeV), and electrons over the energy range ∼25 keV to ∼1 MeV throughout the duration of the mission (2012 – 2019). In this paper we present instrument calibrations, implemented after the Van Allen Probes mission was launched. In particular, we discuss updated rate dependent corrections, possible contamination by “accidentals” rates, and caveats concerning the use of certain products. We also provide a summary of the major advances in ring current science, obtained from RBSPICE observations, and their implications for the future of inner magnetosphere exploration.

## Introduction

The Earth’s ring current has been a subject of interest and considerable study for nearly a century, from the days of ground-based geomagnetism to the satellite era (Daglis et al. 1999; Jordanova et al. 2020). Protons, helium ions, and oxygen ions, of 10 s – 100 s keV, originating both from the solar wind and Earth’s ionosphere, are known to contribute to the ring current species population and energy density content. This is the population that produces equatorial-region depressions in Earth’s magnetic field, during large geomagnetic disturbances, associated with the Dst index (Schmidt 1917; Chapman 1919). The development of the ring current in the inner magnetosphere during geomagnetic storms controls the global electrodynamics of the coupled magnetosphere-ionosphere system. Furthermore, simulations by Ukhorskiy et al. (2015) and comparison with Van Allen Probes storm-time data, demonstrated that the global reconfiguration of the magnetic field due to the ring current development also controls the depletion of radiation belt electrons > MeV, both due to adiabatic depletion at lower L shells, as well as due to magnetopause losses. Therefore, ring current investigations were an essential and integral part of the Van Allen Probes mission.

**Fig. 1:**
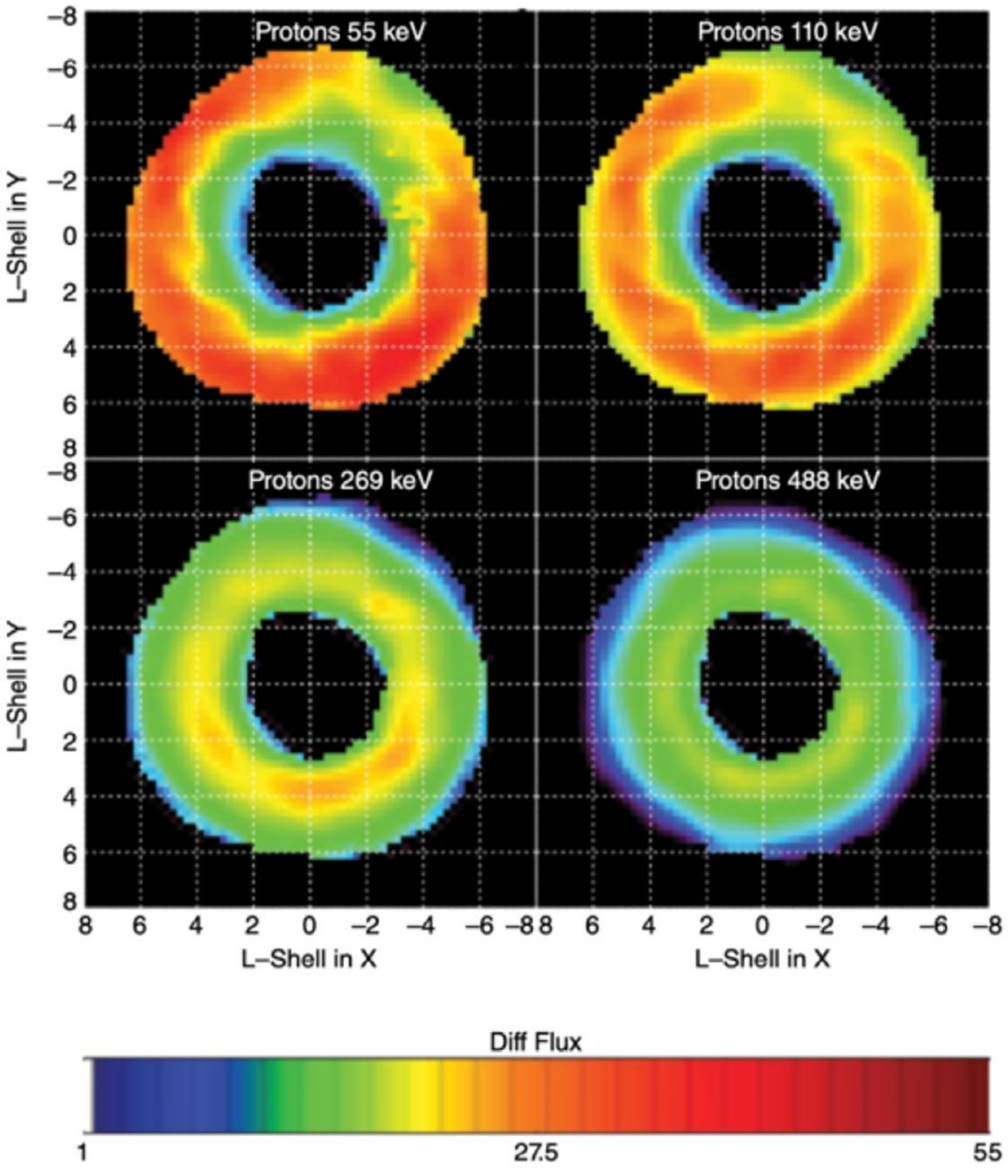
X-Y SM spatial distributions of differential flux [particles/(s ⋅ cm^2^ ⋅ keV)] for protons at energies of (a) 55 keV, (b) 110 keV, (c) 269 keV, and (d) 488 keV. Only “quiet time” data are used here. The flux values are given in decibels (dB = 10*log(differential_flux)). The radial distance is the L-shell, as determined by a simple dipole model, and the SM z-axis points out of the figure (from Lanzerotti and Gerrard 2016)

Comprehensive ring current instrumentation aboard the Van Allen Probes was comprised by three sensors: i) the Radiation Belts Storm Probes Ion Composition Experiment (RBSPICE) (Mitchell et al. 2013) measuring protons (15 – 600 keV), helium ions (65 – 870 keV) and oxygen ions (140 – 870 keV), as well as electrons (25 keV – 1 MeV), ii) the Helium, Oxygen, Proton, and Electron (HOPE) mass spectrometer (Funsten et al. 2013), measuring protons, helium and oxygen ions, as well as electrons (1 eV – 50 keV), and iii) The Magnetic Electron Ion Spectrometer (MagEIS) (Blake et al. 2013; Claudepierre et al. 2021) measuring ions (60 keV – 20 MeV) and electrons (20 keV to 5 MeV). The Van Allen Probes (Mauk et al. 2012) continuously scanned the region inside geosynchronous orbit for ∼ 7 years, and their precession across different MLTs, allowed for long term investigations of the ion composition across the inner magnetosphere, such as the example depicted in Fig. 1, from Lanzerotti and Gerrard (2016).

This paper, is focusing on the RBSPICE instrument, discussing i) in-flight calibrations and lessons learned (Sect. 3) and ii) scientific advances that were enabled by the Van Allen Probes composition data (Sect. 4). This unique, contemporary resource of data provide verifications of, and improvements to, models of ring current ion transport, energization and losses in Earth’s magnetosphere, including their roles in plasma processes for producing instabilities and wave generation (for a recent detailed review of such models please see Toffoletto 2020). A detailed description of the instrument operation, and laboratory calibrations is included in Mitchell et al. (2013). Also, a detailed discussion of the different level products is included in Manweiler et al. (2022). In Sect. 2 of this paper we go over a brief description of the instrument operation, while in Sect. 3 we focus on calibration updates, more specifically, on R_in_ vs R_out_ corrections (Sect. 3.1) and accidentals identification (Sect. 3.2), as well as data caveats (Sect. 3.3). Finally, in Sect. 4, we give a summary of the major advances in the ring current research that have been accomplished in the Van Allen Probes era, and what the implications are for the future of the exploration of the Earth’s inner magnetosphere.

## Principle of Operation

**Fig. 2:**
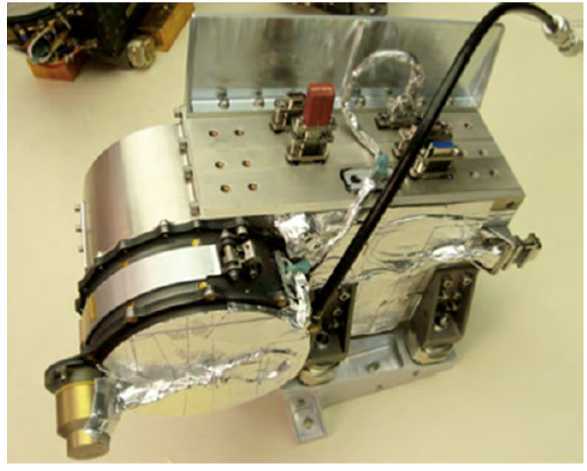
RBSPICE instrument on spacecraft bracket prior to spacecraft integration

**Fig. 3:**
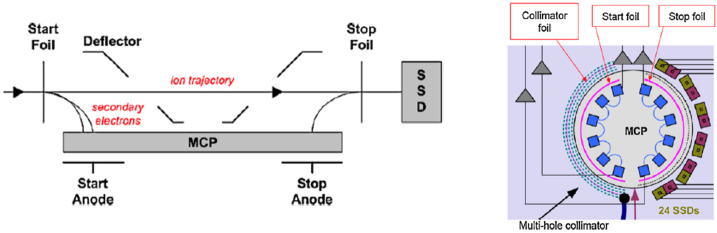
*Left*: Schematic illustration of RBSPICE measurement system; *Right*: Configuration of sensor head (adapted from Mitchell et al. 2013)

RBSPICE, shown in Fig. 2, measures ion energy, direction, and composition using Time-Of-Flight by Energy (*TOFxE*) and Time-of-Flight by Pulse Height (*TOFxPH*) techniques (Mitchell et al. 2013). Figure 3 shows a schematic illustration of the RBSPICE measurement system (left) and the configuration of the sensor head (right).

The RBSPICE sensor head contains a Micro Channel Plate (MCP) detector that measures particle TOF and six Solid State Detectors (SSD) that measure particle energy. The RBSPICE acceptance angle is fan-like, and measures 160 ° by 12 ° with six look directions, each covering 15° along the fan with centers spaced 26.7 ° apart. Due to the fact that the SSDs alternate between ions and (flashed) electrons, there are gaps in the angular coverage between each ion (or electron) look direction. The collimation provided by the collimator restricts the FOV for each SSD to about 15° in the fan direction. Particle direction is determined by the particular SSD pixel in which it is detected as well as the locations of the TOF start and stop pulses. As the ion enters and exits the TOF section of the sensor, it passes through a start and a stop foil respectively, emitting secondary electrons, which are electrostatically guided onto the MCP detector, providing the start and stop signals for the TOF measurements. The energies of the high-energy ions (above ∼50 keV for protons, 65 keV for helium ions and 142 keV for oxygen ions) are measured by the SSDs at the end of the sensor. Event energy and TOF measurements are combined to derive ion mass and to identify particle species (*TOFxE* products). Lower-energy ion intensities (below ∼50 keV for protons) are measured using TOF-only measurements; detection of MCP pulse height, in principle, provides a coarse indication of low-energy particle mass (*TOFxPH* products), although we will mention caveats of that approach later in this manuscript. The sensitivity to ions with energies above the SSD channel thresholds is adjusted automatically by flight software by switching accumulation between the large or small SSD pixels. The total ion geometric factor for RBSPICE is greater than 0.0003 cm^2^-steradian (the large SSD pixels).

Although not a science requirement for RBSPICE, the instrument also has the capability (based on heritage designs) to measure electrons. Energetic electrons from 25 keV to 1000 keV are measured by the electron SSDs. These detectors are covered with 2-μm aluminum metal flashing to keep out protons and other ions with energies less than about 200 keV. No TOF criterion is applied to the electron measurements. Table 1 summarizes RBSPICE performance. For detailed description of RBSPICE’s products and their energy ranges throughout the mission, see Table 5 of Manweiler et al. (2022).

**Table 1:** RBSPICE performance

Parameter	Performance
Electron Energies	25–1000 keV
Ion composition	H: 15–600 keV,
	He: 65–870 keV,
	O: 142–870 keV
Energy resolution	20%
Time sampling	1/36 of the spin (i.e. 0.3 sec)
Angular resolution	15 ° × 12 °
Pitch Angle coverage	0–90 ° or 90 °–180 °
Time for full PA	1 Spin

## In-Flight Calibrations

### R_in_ vs R_out_ Corrections

RBSPICE counts particle events seen by the TOF and SSD systems. The ability of the RBSPICE instrument to count each event is limited by the electronics of the system, causing a failure in counting when counting rates reach approximately 40,000 events per second for the TOF system and 60,000 events per second for the SSD energy mode. When these limits are exceeded, the resulting spectra require scaling, also known as *Rate in* versus *Rate out* (R *vs* R) corrections, to properly represent actual incident event intensities. Corrections are made for various deadtime and veto effects. However, no corrections are made for energy dependent effects such as particle scattering or SSD dead layer.

The original formulae used converting the count-based data products into rates are presented in Sect. 7.2 of Mitchell et al. (2013). In this section, we describe the updated formulae, based on data acquired in-flight. Tables 2, 3, and 4 list the parameters, basic counters, and constants used in the formulae that correct and convert the counters to rates as described in Sects. 3.1.1. – 3.1.3. The basic counters in Table 3 are accumulated and reported in the Basic Rates, that is, products EBR (Electron Basic Rates), IBR (Ion Basic Rates), and ISBR (Ion Species Basic Rates), which are collected for each of the RBSPICE Fast Product Sectors i.e., once every S subsectors (for more details on the data collection patterns and frequency see Manweiler et al. 2022).

**Table 2:** Parameters used in the correction and conversion of the counters to rates. With the exception of the first item, all time intervals are listed in units of the FPGA clock period. Default values are given in square brackets

Parameter	Description
Clk_period_	Period of clock used in event logic [100 ns]
Max_IDLE_	Interval during which counts are accumulated [duration/(10^−9^*Clk_period_)]
ST_dead_	Start counter deadtime due to synchronization logic [2]
ST_miss_	Interval during which Starts cannot be detected by the State Machine [2]
SP_dead_	Stop counter deadtime
SP_veto_	Interval during which additional SP pulses cause the event to be discarded [2]
Rdt_veto_	Interval for inhibiting ST and SP counters during TOF chip reset [1]
PUR_veto_	Interval during which a second SSD pulse causes the event to be discarded [24]
PKD_reset_	Interval for resetting the Peak-Detector [4]
PKD_dt_	Minimum duration of Peak-Detector busy [17], (busy extended for >1 MeV)

**Table 3:** Basic counters

Counter	Description
Start0	Counts pulses on the end of the Start Anode delay line nearest to look direction 0
Stop0	Counts pulses on the end of the Stop Anode delay line nearest to look direction 0
TOFCoin	Counts Start-Stop pairs, Start before Stop, within a maximum TOF window
PulseHeight	Counts PH events from the Start Anode. Slower circuit than Start0, and required for Valid TOFxPH event, leading to need for R *vs* R correction using this term.
SSD*i*	Counts pulses on SSD of look direction *i*, *i* = 0,1,2…5.
SSD*i*_dt_	Counts clock periods that SSD*i* is above threshold and blind to additional pulses.
IDLE	Counts clock periods that Event State machine is free and able to accept new events
Valid_TOFxE_	Counts coincident Start, Stop and SSD pulses satisfying valid-event criteria.
Valid_TOFxPH_	Counts coincident Start, Stop and MCP PH pulses satisfying valid-event criteria.
Valid_Energy_	Counts SSD pulses satisfying valid-event criteria.
Valid_Proc_	Counts valid events processed by the software.
Rdt	Counts the number of TOF chip resets.

**Table 4:** Constants used in the Ion Species Mode Spectra conversion to Rates

Constant	Value
K_1E_	0.3
K_1PH_	0.15
K_2PH_	0.15
C_SSD_	2 FPGA clock ticks
C_PH_ [for S/C A]	0.86
C_PH_ [for S/C B]	0.775

#### Correction and Conversion of Singles Counts to Rates

The corrections detailed below are non-linear combinations of the counters and therefore the integration period should be short enough that the counts per sector are reasonably constant. Please note that in all the equations below the $Clk_{period}$ parameter is in ns, so a factor of 10^−9^ needs to be applied in order for the rates to be *per second*.

*Correction of Start and Stop Anode Rates*: The Start0 and Stop0 pulses, as defined in Table 3, have a fixed deadtime. Also, counting is inhibited during TOF chip resets. This means that the true number of pulses is diminished by the average number of pulses occurring during these deadtimes. Therefore, the correction from raw counts to anode rate is given by equations:

1 $$\begin{aligned} Start_{rate} & = \frac{{Start0} / {\left ( Max_{IDLE} * Clk_{period} \right )}}{1- \left ( {Start0* ST_{dead}} / {Max_{IDLE}} \right ) - \left ( {Rdt* Rdt_{veto}} / {Max_{IDLE}} \right )} \end{aligned}$$

2 $$\begin{aligned} Stop_{rate} & = \frac{{Stop0} / {\left ( Max_{IDLE} * Clk_{period} \right )}}{1- \left ( {Stop0* SP_{dead}} / {Max_{IDLE}} \right ) - \left ( {Rdt* Rdt_{veto}} / {Max_{IDLE}} \right )} \end{aligned}$$

*Correction of SSD Rates*: The pulse from the Peak Detector (PKD) leading-edge discriminator for $SSDi$ has a deadtime equal to the time over threshold which is variable, being longer for larger pulses. Because of the variable deadtime the rate is determined by using the observation that the average time for a pulse to occur starting from an arbitrary point is equal to the reciprocal of the rate. Therefore the correction from raw counts to rate is given by

3 $$\begin{aligned} SSDi_{rate} = \frac{SSDi}{\left ( Max_{IDLE} - SSDi_{dt} \right ) * Clk_{period}} \end{aligned}$$

#### Correction and Conversion of Energy Mode Spectra to Rates

The anode and SSD rates described above are integrated into the calculation of rates to adjust the Energy Mode spectra counts, due to the inability of the system to identify all incident events. The rate in bin $ij$, where $i\in \left \{ 0...5 \right \}$ are the look directions, and $j\in \left \{ 0... n_{ch} \right \}$ are the energy channels ($n_{ch}$ is the number of energy channels for the particular data product), is calculated as

4 $$ R_{ijk} = \frac{h_{ij}}{Valid_{Proc}} * \frac{Valid_{Energy}}{IDLE* M_{k} * Clk_{period}} * C_{PKD_{reset}}^{i} * C_{PUR_{veto}}^{i} $$

where $h_{ij}$ are the L0 counts in bin $ij$, and $M_{k} = 1$, N1, N1*N2*Z for $k = 1$ (Fast products), 2 (Medium products), 3 (Slow products). For details on the sector/subsector scheme see Manweiler et al. (2022). $IDLE* M_{k}$ can be replaced by $\sum IDLE$, i.e. the sum of the $IDLE$ values over the time during which the $h_{ij}$ are accumulated. The first term in Eq. (4) is the fraction of processed events found in bin $ij$, which should be identical to the fraction of *Valid*_*Energy*_ events which would be assigned by the software to bin $ij$ if the software could handle the full rate (this is because *Valid*_*Proc*_ counts are events processed by the software, even those that fail software selections). The second term is the *Valid*_*Energy*_ rate corrected for deadtime of the Event state machine. The Event state machine deadtime includes a contribution from the Peak-Detector (PKD) state machines which feed it. Each PKD state machine (one for each SSD) becomes busy when its input pulse exceeds threshold and remains busy for an interval of clock periods given by max(TOT,17) where TOT is the time-over-threshold expressed in clock periods. After this interval the Event state machine becomes again live while the PKD is reset for 4 clock periods. Subsequent pulses on this SSD occurring during the reset period are ignored, that is they do not cause new events to be initiated. The third term in Eq. (4) corrects for this loss of events by the reciprocal of the probability of having no pulses in the PKD reset interval and is given by

5 $$ C_{PKD_{reset}}^{i} = \exp \left ( SSDi_{rate} * PKD_{reset} * Clk_{period} \right ) $$

A further loss of events occurs if one or more additional SSD$i$ pulses are detected within 7 clock periods after the initial pulse. Such pile-up events are discarded by the Event state machine to avoid recording an erroneous pulse height measurement. The last term in Eq. (4) corrects for this effect and is given by

6 $$ C_{PUR_{veto}}^{i} = \exp \left ( \frac{SSDi * PUR_{veto}}{Max_{IDLE}} \right ) $$

The reason for using the raw $SSDi$ counter (rather than corrected rate) in Eq. (6) is that pile-up is detected only if the initial pulse falls below threshold before a subsequent pulse arrives, an effect which is incorporated in the raw counter behavior but removed in the corrected rate (Eq. (3)).

#### Correction and Conversion of Ion Species Mode Spectra to Rates

In this section we describe the correction for the Ion Species mode measurements. Again the counts are accumulated in a two dimension array of look directions, $i$, and “energy bin”, $j$, which is actually the *TOFxE* and *TOFxPH* ID generated by the event processor for each incident event.

The conversion from *TOFxE* spectrum to rates is then given by

7 $$\begin{aligned} R_{\left ( TOFxE \right ) ij} = {}&\frac{h_{\left ( TOFxE \right ) ij}}{Valid_{Proc}} * \frac{Valid_{TOF\times E} + Valid_{TOF\times PH}}{ IDLE* M_{k} * Clk_{period}} * C_{PKD_{reset}}^{i} * C_{PUR_{veto}}^{i} \\ &{} * \exp \left ( \frac{Stop0* SP_{veto}}{Max_{IDLE}} \right ) * \exp \left ( \frac{Start0* ST_{miss}}{Max_{IDLE}} \right ) \\ &{} * (Valid_{TOF\times E} +VE_{correction} )/ Valid_{TOF\times E} \end{aligned}$$

where

$$\begin{aligned} VE_{correction} ={}& K_{1E} * TOFCoin \\ &{}* \left ( \frac{Valid_{TOF\times E}}{Valid_{TOF\times E} + Valid_{TOF\times PH}} * \left \{ 1-exp \left ( - C_{SSD} * \frac{\sum SSDi}{Max_{IDLE}} \right ) \right \} \right ) \end{aligned}$$

The conversion from *TOFxPH* spectrum to rates is given by

8 $$\begin{aligned} R_{\left ( TOFxPH \right ) ij} ={}& \frac{h_{\left ( TOFxPH \right ) ij}}{Valid_{Proc}} * \frac{Valid_{TOF\times E} + Valid_{TOF\times PH}}{ IDLE* M_{k} * Clk_{period}} * \exp \left ( \frac{Stop0* SP_{veto}}{Max_{IDLE}} \right ) \\ &{}* \exp \left ( \frac{Start0* ST_{miss}}{Max_{IDLE}} \right ) * (Valid_{TOF\times PH} +VPH_{correction1} \\ &{}+ VPH_{correction2} )/ Valid_{TOF\times PH} * PH_{corr} \end{aligned}$$

where

$$\begin{aligned} &VPH_{correction1} \\ &\quad = K_{1PH} * TOFCoin * \left ( \frac{Valid_{TOF\times PH}}{Valid_{TOF\times E} + Valid_{TOF\times PH}}\right. \\ &\left.\qquad {} * \left \{ 1-exp \left ( - C_{SSD} * \frac{\sum SSDi}{Max_{IDLE}} \right ) \right \} \right ) \end{aligned}$$

$$\begin{aligned} &VPH_{correction2} \\ &\quad = K_{2PH} * TOFCoin * \left ( \frac{Valid_{TOF\times PH}}{Valid_{TOF\times E} + Valid_{TOF\times PH}} \right.\\ &\left.\qquad {} * \left \{ 1-exp \left ( - C_{SSD} * \frac{\sum SSDi}{Max_{IDLE}} \right ) \right \}^{2} \right ) \end{aligned}$$

and

$$ PH_{corr} = \mathrm{C}_{\mathrm{PH} \left ( \mathrm{A},\mathrm{B} \right )} * \frac{Start0}{PulseHeight} $$

Similarly to Eq. (4), $IDLE* M_{k}$ in Eqs. (7), and (8) can be replaced by $\sum IDLE$.

Following, we explain the extra terms that show up in Eqs. (7) and (8), compared to Eq. (4).

The event logic requires no Stop hit in the two clock periods ±2 from the triggering clock period (note that the clock periods adjacent to the triggering period are guaranteed to be empty by the synchronization logic). The $\exp \left ( \frac{Stop0* SP_{veto}}{Max_{IDLE}} \right )$ factor corrects for those non-isolated Stop hits.

The deadtime of the foreground hits (i.e. coincident with TOF hits) is at least partially accounted for by the Event state machine IDLE counter. The presence of background will lead to an inefficiency of triggering *TOFxE* events. This also applies to the effects of foreground, in the sense that the system is Start-triggered, and while it is busy processing an event, it will ignore additional Start hits (they will not trigger the State Machine, and they will be unknown to the FPGA). Whether a consequence of high background rates, high foreground rates, or both, the $\exp \left ( \frac{Start0* ST_{miss}}{Max_{IDLE}} \right )$ factor corrects for missed Starts due to State Machine dead time.

The terms $VE_{correction}$ in the *TOFxE* product, and $VPH_{correction1}$, and $VPH_{correction2}$ in the *TOFxPH* product correct for events rejected by the FPGA when two (or more) SSDs are triggered during the analysis of a valid TOF event, leaving the FPGA unable to render a unique valid event. These terms become important when the rates are particularly high. The reason that an analogous correction must be made to the *TOFxPH* rates at times of high SSD rates is because events with *valid* *TOFxPH* that also have a coincident hit on an SSD, which does not correspond to the TOF Stop position, are rejected by the FPGA logic without triggering a counter.

**Fig. 4:**
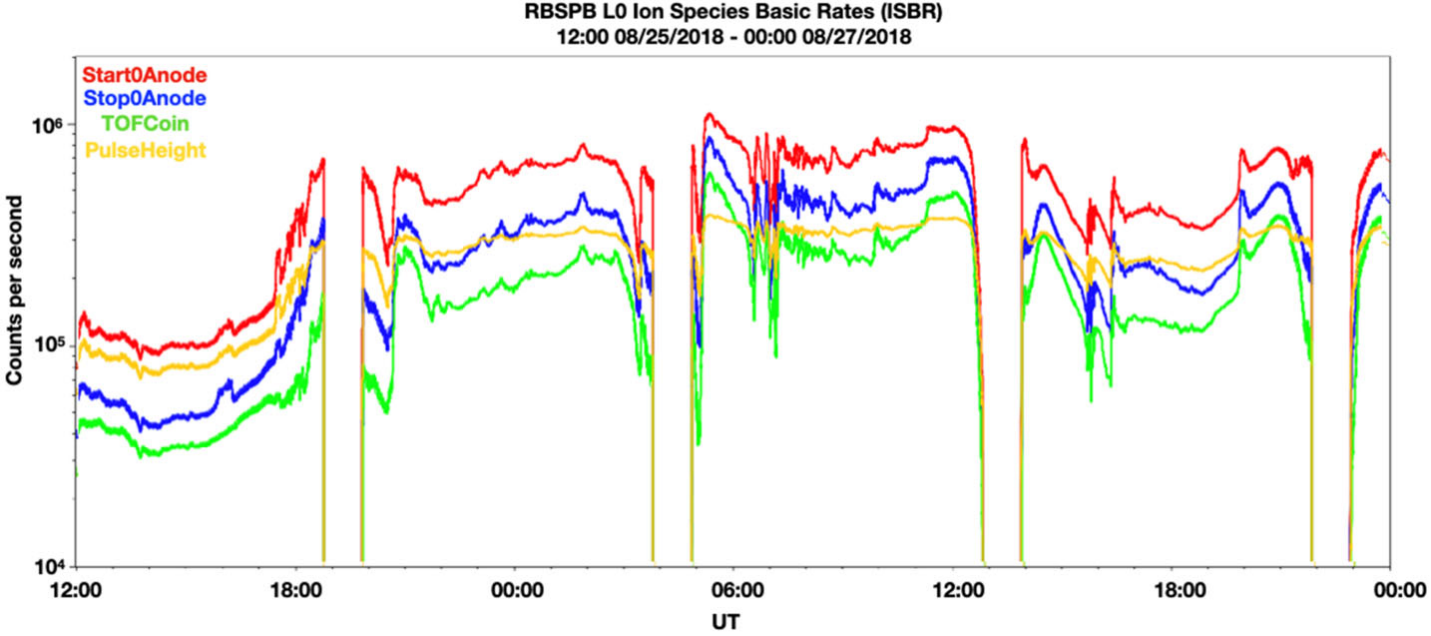
Counts per second for *Start0*, *Stop0*, *TOFCoin*, and *PulseHeight*

The $PH_{corr}$ term in the *TOFxPH* product accounts for the fact that to be counted as valid, an event must not only produce a Start event, but must also produce a pulse height in the MCP start area above a trigger threshold. The $PH$ circuit, an energy ASIC (Application Specific Integrated Circuit), integrates the charge deposited on the anode, thus, it is a slower circuit than the *Start0* CFD (Constant Fraction Discriminator) counter. As a result, at high rates, the $PH$ counter will begin to miss events that the Start circuit can still resolve. So while the TOF is produced by a Start-Stop pair, the requirement for a $PH$ to also be triggered reduces the *valid* TOF rate by the ratio *PulseHeight*/*Start0*. The correction for this threshold effect is therefore the reciprocal, *Start0*/*PulseHeight*, times a constant that reflects slight differences in the pulse height threshold levels set for RBSPICE A and B. Because a $PH$ measurement is required to form a valid *TOFxPH* event (but NOT to form a valid *TOFxE* event), science products that are derived from valid *TOFxPH* events have the additional correction factor $PH_{corr}$ must be applied. Figure 4 displays counting rates (not *R vs R* corrected) for $Start0$, $Stop0$, *TOFCoin*, and $PulseHeight$ preceding and during the August 25–26 2018 storm. Note that when the rates get very high, the $PulseHeight$ does not display the same dynamic range as Start0 since, as explained earlier, the PH circuit is slower than the Start0 one.

In order to verify the accuracy of our *R vs R* corrections for the *TOFxE* and *TOFxPH* products, we need to satisfy the following equation (assuming that the singles rates, which have much faster characteristic response times, are unaffected by rate-associated reductions):

9 $$ \sum _{i} \frac{SSDi}{GF_{i} * Eff_{s}^{scat} * Max_{IDLE}} = \sum _{i} \sum _{j} \frac{h_{ijs}}{GF_{i} * Eff_{js}} * \left \{ R\textit{ vs }R \right \} $$

Where:

- $GF_{i}$ is the geometric factor for the $i$th telescope;
- $Eff_{s}^{scat}$ is the ion species efficiency (≤1) from scattering in the collimator and Start foils (assumed to be dominated by protons);
- $h_{ijs}$ is the counts in each energy bin (j) for each telescope (i) for the species (s) (generally either *TOFxE* Ions or *TOFxE* H);
- $Eff_{js} =\ Eff_{js}^{scat} *\ Eff_{js}^{TOF}$ for each energy (j) and species (s); note that $Eff_{js}$ and $Eff_{js}^{scat}$ are explicitly known, while $Eff_{js}^{TOF}$ is not.
- $Eff_{js}^{TOF}$ is the *TOF* efficiency for that energy and species (we assume protons for *TOFxE* Ions),
- $\left \{ R\ vs\ R \right \}$ is the entire set of factors that correct the binned counts. For example, for the *TOFxE* products: $\left \{ R\ vs\ R \right \} = \frac{1}{Valid_{Proc}} *\ \frac{Valid_{TOF\times E} \ + Valid_{TOF\times PH}}{\ IDLE* M_{k} * Clk_{period}} * C_{PKD_{reset}}^{i} * C_{PUR_{veto}}^{i} * \exp \left ( \frac{Stop0* SP_{veto}}{Max_{IDLE}} \right ) *exp \left ( \frac{Start0* ST_{miss}}{Max_{IDLE}} \right ) * (Valid_{TOF\times E} +VE_{correction} )/ Valid_{TOF\times E}$

This equation basically states that the sum of the events measured by the SSDs (when properly adjusted for geometric factor and reduced efficiency associated with scattering of ions in the foils) should equal the sum of the counts analyzed as *TOFxE* events, when they are adjusted by their own geometric factors and efficiencies, and corrected for dead-time and other rate-associated adjustments associated with front-end processing in the FPGA. Assuming the $GF_{i}$ are independent of $i$, and $Eff_{s}^{scat}$ is also independent of $i$, this equation can be simplified to:

10 $$ \sum _{i} \frac{SSDi}{ Max_{IDLE}} = \sum _{i} \sum _{j} \frac{h_{ijs} * Eff_{s}^{scat}}{Eff_{js}} * \left \{ R\textit{ vs }R \right \} $$

**Fig. 5:**
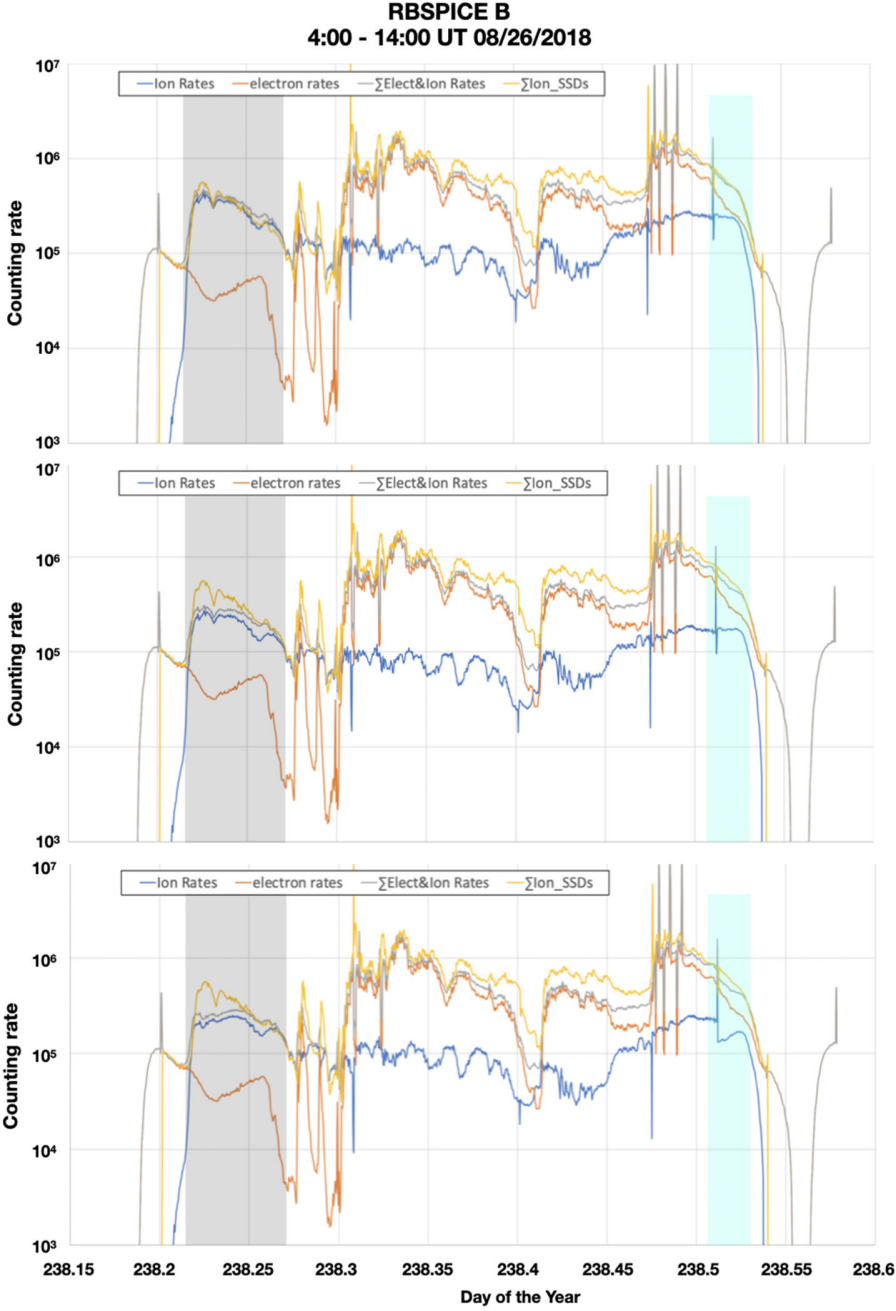
*Top panel:* *R vs R* Corrected *TOF* *Ion* rates according to Eq. (7) (blue), electron rates (orange), sum of SSD ion rates (yellow), and sum of electron and *TOF* *Ion* rates (grey); *Middle panel*: same as top panel but $\boldsymbol{ST}_{\boldsymbol{miss}} = \mathbf{0}$ in Eq. (7); Bottom panel: same as top panel, but $\boldsymbol{ST}_{\boldsymbol{miss}} = \mathbf{0}$ and $\boldsymbol{VE}_{\boldsymbol{correction}} =\boldsymbol{0}$ in Eq. (7)

Figure 5 demonstrates how using the correct set of *R vs R* corrections, satisfies Eq. (10). All three panels show corrected TOF required Ion (TOF Ion) rates in blue, electron rates in orange, sum of SSD ion rates (no TOF measurement) in yellow, and sum of electron and TOF Ion rates in grey, on August 26 2018 from 4:00 – 14:00 UT. However, in each panel a different correction has been applied to the TOF Ion rates: i) In the top panel Eq. (7) is used, ii) in the middle panel, the $ST_{miss}$ parameter in Eq. (7) is set to 0, and iii) in the bottom panel, both $ST_{miss}$ parameter and $VE_{correction}$ term are set to 0. When the appropriate correction factor is used (top panel), the TOF Ion rates match the sum of SSD Ion rates, in the time interval when the electron rates were much lower than the TOF Ion rates (shaded in grey), and thus the SSD ion measurements (no *TOF* required) were not contaminated by electrons (see discussion in Sect. 3.3 below). Note that at later times, when the electron rates are overwhelming the SSD measurement, a comparison between *TOF* *Ion* rates and sum of SSD ion rates is not possible due to contamination of the SSD ion rates by electrons. Therefore, by using the appropriate *R vs R* correction to the *TOF* *Ion* rates, Eq. (10) is satisfied. On the contrary, when $ST_{miss} =0$ (middle panel), that is, there is no correction for missed Start counts due to State Machine dead time, the *TOF* *Ion* rates do not match the SSD ion rates in the grey shaded time interval. When both $ST_{miss} =0$ and $VE_{correction} =0$ (bottom panel) then not only do not the *TOF* *Ion* rates match the sum of SSD ion rates in the grey shaded interval, but also *TOF* *Ion* rates do not match across rate-driven switch from small to large pixels (cyan shaded time interval).

**Fig. 6:**
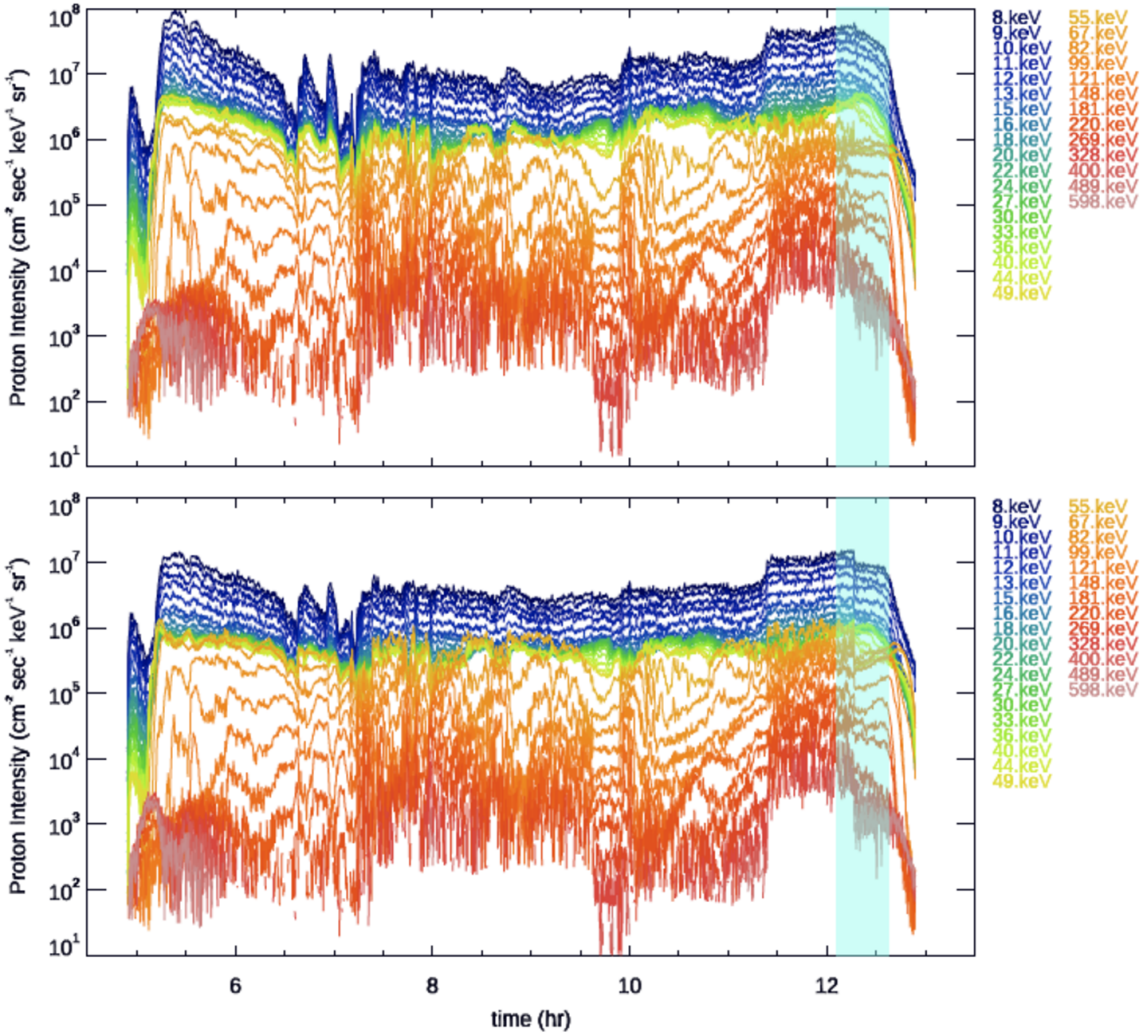
Proton intensities (*TOFxE* product for energies ≥ 55 keV and *TOFxPH* product for energies $\boldsymbol{\leq} $ 49 keV), for the same time interval as in Fig. 5, corrected using Eq. (7) (top panel), and corrected using Eq. (7) with $\boldsymbol{ST}_{\boldsymbol{miss}} = \mathbf{0}$ and $\boldsymbol{VE}_{\boldsymbol{correction}} =\boldsymbol{0}$ (bottom panel)

Figure 6 shows proton intensities (*TOFxE* product for energies ≥ 55 keV and *TOFxPH* product for energies ≤ 49 keV), for the same time interval as in Fig. 5, corrected using Eq. (7) (top panel), and corrected using Eq. (7) with $ST_{miss} = \mathbf{0}$ and $VE_{correction} =0$ (bottom panel).

As it can be seen, by not using the appropriate R vs R corrections, the absolute value of proton intensities decreases, and there is a mismatch in the intensities during the switch from small to large pixels (cyan shaded time interval).

### Accidentals

As it was already briefly mentioned in Sect. 2, the RBSPICE instrument uses a foil to generate secondary electrons as the measured ions enter through the entrance collimator (Start foil), and another foil just before they reach the SSDs that register their energies (Stop foil). The instruments include electrostatic configurations that guide those secondary electrons to Start and Stop areas on an MCP, registering timing pulses for determining the particle velocity. Various other environmental sources (UV, low energy plasma, electrons) can also generate secondary electrons on these foils, contributing to the rates of Start and Stop pulses registered by the MCP. The particles of interest are identified by logic which requires that a Start pulse be followed by a Stop pulse within a defined maximum time window of ∼150 ns (deemed a “valid” time of flight, or *TOF*). For higher energies (sufficient to register above the energy thresholds of the SSDs), the valid *TOF* must also be accompanied by an energy signal in the SSD within a longer event window, and the positions of the Start, Stop, and energy pulses must be consistent with a straight particle trajectory across a sensor diameter.

Events known as “accidentals” are cases in which the valid event criteria (Start followed by Stop and, for higher energies, an accompanying energy measurement with the required positions satisfied) are met by randomly generated Start, Stop, (and energy, for particles that also hit the SSDs) pulses that happen to “accidentally” meet the valid event criteria. Generally, for valid event criteria that do not require energy (i.e. *TOF* Ions, or *TOFxPH* protons), the rate of accidentals can be calculated by $R_{\textit{accidental}(\textit{TOF})} = R_{\textit{start}} \times R_{\mathit{stop}} \times \textit{TOF}_{\textit{max-window}}$, where *TOF*_*max-window*_ = 150 ns. The contribution to any particular energy channel is $R_{\textit{accidental}(\textit{TOF})} \times \textit{TOF}_{\textit{bin-bandwidth} }/ \textit{TOF}_{\textit{max-window}}$, because the accidental TOF must fall within the channel bandwidth to contribute to that *TOF* bin. This rate is further reduced by a factor of 6 in the case of RBSPICE, because each of the 6 start positions is required to match its corresponding stop position for validity. For *TOFxE* events, the accidental rate is $R_{\textit{accidental}(\textit{TOFxE})} = R_{\textit{start}} \times R_{\textit{stop}} \times\textit{TOF}_{\textit{max-window}} \times R_{\mathit{ssd}}\times T_{\mathit{ssd}}$ where $T_{\mathit{ssd}}$ is the characteristic time width of the SSD energy measurements (∼ 1 μs). This rate is further reduced by a factor of 1/36 by requiring that the Start, Stop, and SSD positions coincide. And it is reduced even further by requiring that the *TOF* be consistent with the $E$ for a H, He, or O (that is, the velocity v determined by the *TOF* must correspond to the measured $E$ deposited in the SSD such that $E = 1/2\text{ mv}^{2}$). For these reasons, accidentals are typically negligible for *TOFxE* species products, even during times with rates sufficiently high to produce large numbers of *TOFxPH* accidentals.

**Fig. 7:**
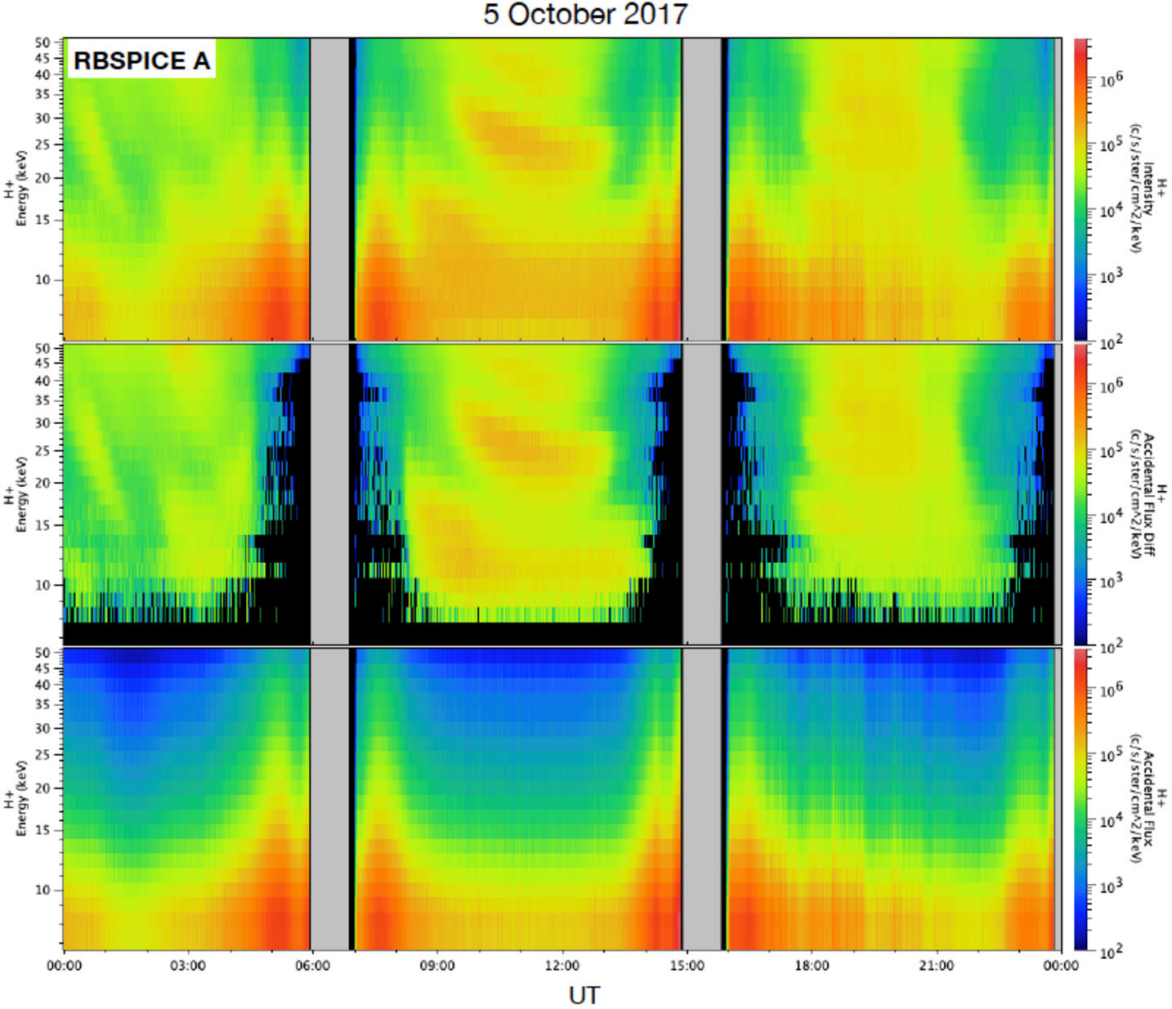
*Top panel*: TOFxPH proton intensity; *Bottom panel*: accidentals rate $R_{\mathit{accidental}\ (\textit{TOF})}$; *Middle panel*: TOFxPH proton intensity after the accidental count rates have been subtracted, for RBSPICE A during 5 October 2017

**Fig. 8:**
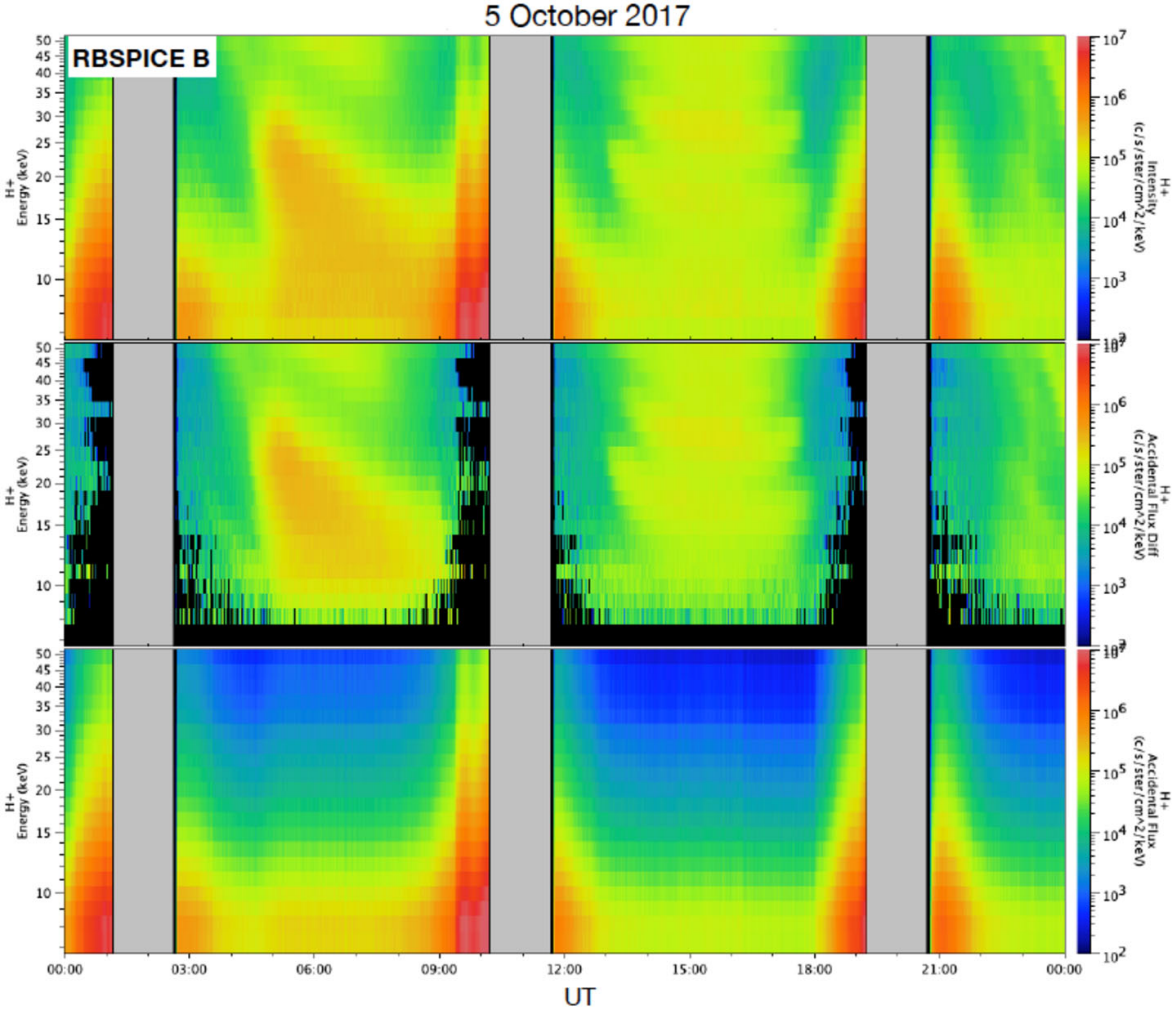
Same as Fig. 7 but for RBSPICE B

When the Start, Stop, (and energy) rates are low, the probability of generating accidentals is low. When rates become very high, $R_{\mathit{accidental}}$ can become significant, and at times can even dominate the events that the logic deems as valid. For RBSPICE, this condition occurs for particularly high MCP Start and Stop rates that often manifest near perigee. The causes of these high rates are not fully understood, but they are a very repeatable feature in the RBSPICE data. Because of differing sensitivity to environmental conditions, the high MCP rates under otherwise identical conditions are not identical for RBSPICE A and B. Figures 7 and 8 provide examples from 5 October 2017 that illustrate the effect that accidentals have on the *TOFxPH* protons. The top panel in both figures shows the *TOFxPH* proton intensity, the bottom panel shows the accidentals rate $R$_*accidental* (*TOF*)_, and the middle panel shows the *TOFxPH* proton intensity after the accidental counts have been subtracted. As it can be seen accidentals dominate the *TOFxPH* proton intensities for energies below ∼15 keV around apogee and even for higher energies around perigee.

### RBSPICE Product Caveats

In Sect. 3.2. we described in detail the possible contamination of the RBSPICE products from accidentals. In this section, we present caveats that need to be taken under consideration when analyzing certain RBSPICE products.

*TOF required products*: RBSPICE has experienced challenges with micro-discharges when taking measurements close to perigee. Because of that, the high voltage supply was turned off within L ∼ 3 (slightly different for A and B spacecraft) and thus no TOF required products are available for low L-shells. As the mission progressed the RBSPICE discharge problems diminished, and the L shell at which the *TOF* products were suppressed was lowered further, in some instances riding through perigee, but usually excluding L-shells below 2.5 (more on the topic of micro-discharges on “puck” type energetic particle detectors was discussed by Clark et al. (2016).

*ISRHELT* (*Ion Spectra - High Energy Low Time Resolution*): With respect to the ISRHELT product, users should be aware that, although this is identified as an “Ions” product, it is contaminated by electrons most of the time. This is because this product is taken during the “energy” mode of the instrument, i.e., there is no *TOF* information assigned to it, but only energy deposition to the SSDs. Alternatively, the *TOFxE_Ion* product should be used to analyze *TOF*-triggered ion events (no composition information), or the *TOFxE*[$H$, $He$, $O$] products for high energy composition and *TOFxPHH* product for low energy protons.

**Fig. 9:**
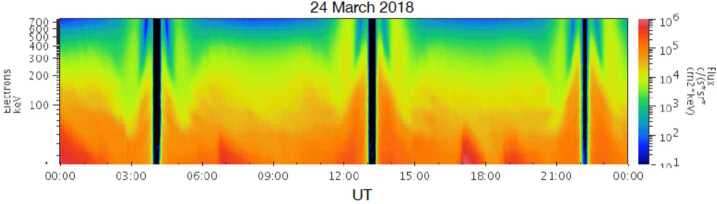
RBSPICE Electrons intensities during 24 March 2018

*ESRHELT* (*Electron Spectra - High Energy Low Time Resolution*): Although not a science requirement for RBSPICE, the instrument also has the capability to measure electrons in the energy range of 25 keV to 1 MeV. However, it should be noted that electron spectra of energies above ∼250 keV (see Fig. 9) are contaminated by penetrating radiation belt electrons of energies above 1 MeV, as well as by energetic protons that penetrate their aluminum flashing that stops protons below ∼400 keV.

*TOFxPH_O* (*TOFxPH Oxygen*): Although oxygen does produce a statistically higher MCP pulse height than hydrogen, the spread in pulse heights is large for both species, and as a result, the high pulse height tail of the hydrogen distribution significantly overlaps the mean of the oxygen distribution, while the low pulse height tail of the oxygen distribution lies on top of the mean pulse height of the hydrogen distribution. Therefore, with the potential for counts from either species contributing significantly to counts assigned to the other, the separation of species is poor. For example, if a pulse height threshold is set as a discriminator between oxygen and hydrogen, in an environment entirely dominated by hydrogen the high pulse height tail of the hydrogen distribution will be interpreted by the onboard software as oxygen. Likewise, in an environment dominated by oxygen, the low pulse height tail of the oxygen will be interpreted as hydrogen. Since the Earth’s magnetosphere is a hydrogen dominated environment for the most part, the *TOFxPH_O* product is mostly contaminated by hydrogen, and thus should not be used as a low energy oxygen product. Unlike the RBSPICE instrument, the pulse height distributions of protons and oxygen were well separated from each other in Energetic Neutral Atom cameras, like IMAGE/HENA and Cassini/INCA, where the ENAs hit the MCPs directly, producing a more statistically well-defined pulse height. Therefore it is likely that the poor separation in RBSPICE is due to the fact that the primary particle does not impact the MCP directly, but instead it is the pulse height resulting from the number of secondary electrons generated by the ion/foil interaction that is being measured. And the resulting distributions for H and O at a given velocity are characterized by Poisson statistics for small numbers, resulting in ambiguity and overlap.

## Scientific Advances

The Van Allen Probes highly elliptical, equatorial orbit with apogee within geosynchronous orbit (5.8 R_E_) allowed for continuous sampling of the inner magnetosphere, which was crucial in capturing the transport, acceleration, and loss of the ring current ions, especially during highly dynamic events, such as geomagnetic storms (with ∼9 hr orbit, the Van Allen Probes was sampling the same region at least 2-3 times during the course of a geomagnetic storm). That continuous sampling, in combination with the high temporal (∼14 sec for a full distribution) and energy resolution of the RBSPICE instrument, led to a wealth of studies, showcasing, for the first time, the abundance of dynamic, localized in MLT, energetic ion injections, well within geosynchronous orbit, during geomagnetically active times. Although several studies in the past highlighted that this, so-called mesoscale mode of particle transport and acceleration is ubiquitous in the tail and plasma sheet (e.g. Runov et al. 2011; Gabrielse et al. 2014), the Van Allen Probes era studies, using the RBSPICE data, revealed how ion injections in the inner magnetosphere could be significant contributors to the buildup of the ring current during geomagnetic storms. Furthermore, the composition data, allowed for detailed studies of how different ion species get accelerated within the inner magnetosphere and how energy gets transferred between those ion species and electromagnetic waves.

In the next sections we summarize the above mentioned findings of various studies that used RBSPICE data or a combination of RBSPICE and HOPE data, in order to address major issues of the ring current ion dynamics. A very comprehensive review of the ring current observations in the last 50 years, including the latest investigations during the Van Allen Probes era can also be found in Kistler (2020).

### Ring Current Dynamics

One of the major questions that has been under debate for a long time is what role, if any, the mesoscale energetic particle injections play in the buildup of the ring current during geomagnetic storms. Gkioulidou et al. (2014), analyzing RBSPICE proton data from ∼20 – 600 keV, showed that multiple small-scale injections occurred during the main phase of the March 17th 2013 geomagnetic storm ($\mathrm{Dst}\sim -137\text{ nT}$), as earthward as L = 4. Although isolated injections have been previously reported inside geosynchronous orbit, the large number of small-scale injections observed in this event suggests that, during geomagnetic storms injections provide a robust mechanism for transporting energetic ions deep into the inner magnetosphere. Based on individual injection properties, such as associated pressure enhancement, the time duration of this enhancement, and the lowest and highest energy channels exhibiting a sharp increase in their intensities, the authors estimated that these small-scale injections contribute ∼ 30% of the total energy gain in the storm time inner magnetosphere, however it should be noted that contribution of oxygen ion injections was not included in this study. Menz et al. (2016), examined the pressure enhancements during the same geomagnetic storm, including both oxygen and protons in their analysis. By using Weimer 1996 electric field, they modeled particle drift trajectories and showed that the key features of the temporal development and local time dependence of the pressure peak during the storm can be explained by variations in the open/closed drift path boundary with local time or due to temporal changes in the electric field that occur between one observation and the next. Therefore they concluded that the pressure buildup can be attributed to large scale convective transport of the near-Earth plasma sheet source into the inner magnetosphere, in agreement with previous studies (e.g., Jordanova 2005 and references therein).

The following modeling studies, which were published in parallel with the above observational studies, contributed further to understanding the relative importance of the quasi-steady, large-scale convective transport vs mesoscale, dynamic transport. Yang et al. (2015), modeled the main phase of 20 idealized geomagnetic storms using RCM-E, an inner magnetosphere model that incorporated self-consistent magnetic and electric fields. They showed that particles injected within depleted flux tube mesoscale “bubbles”, traveling earthward due to interchange instability, are the major contributor to the plasma energy inside geosynchronous orbit for storms with $\text{Dst} < -70\text{ nT}$, while nonbubble-like transport from the plasma sheet and the trapped particles only contribute ∼20% on average. The conclusion of their study was, therefore, that the plasma sheet bubbles are the dominant source of the ring current for moderate and intense storms. In a follow up study, Yang et al. (2016) conducted RCM-E numerical experiments, in order to understand why stand-alone ring current models have been successfully producing storm time pressure enhancements without specifying explicit localized transient injections along their outer boundaries (at geosynchronous orbit), even though observations and simulations have suggested that bursty bulk flows and associated particle injections can have a substantial contribution to the storm time ring current energy. They showed that, even though the observed fluctuations in the plasma population and electric field can only be captured when the mesoscale injections are included in the simulation, when the bubble effects are being smoothed out at the geosynchronous boundary the model can predict approximately the same large-scale pressure distribution. Therefore, the authors concluded that, although bubble injections are the main source of the ring current during storm main phase, quasi-steady convective models with their boundaries set at geosynchronous orbit are able to predict the large-scale buildup of the ring current pressure distribution, given the appropriate conditions along that boundary, which reflect integrated effect of injections.

The above investigations indicate that, despite the significant progress achieved during the Van Allen Probes era, estimating with in-situ observations to what extend the mesoscale transport and acceleration mode is the main contributor to the buildup of the energy density in the storm-time, inner magnetosphere is a major challenge.

**Fig. 10:**
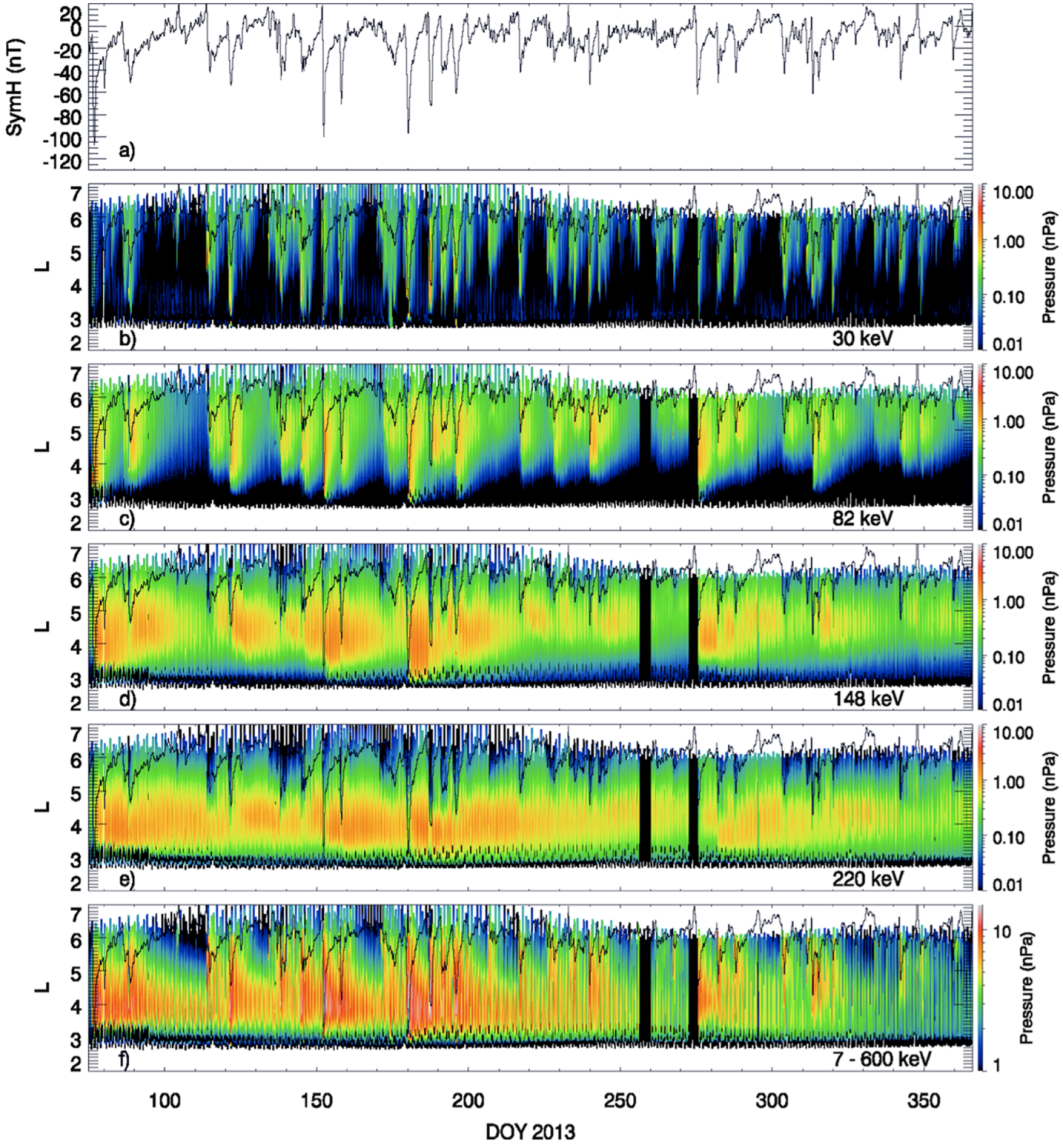
(a) SYM-H index acquired by the OMNIweb service from 18 March to 31 December 2013, partial perpendicular pressures along Van Allen Probe B orbit, contributed by protons of (b) 30, (c) 82, (d) 148, and (e) 220 keV energies for the same time interval, (f) perpendicular pressure contributed by 7–600 keV energy protons for the same time interval (from Gkioulidou et al. 2016)

Several Van Allen Probes studies have focused on the relative contribution of different energies to the ring current pressure during the course of a geomagnetic storm. Gkioulidou et al. (2016) investigated the long-term ring current proton pressure evolution in Earth’s inner magnetosphere and found that proton dynamics, and the resulting energy budget in the inner magnetosphere, do not vary strictly on storm time timescales as those are defined by the Dst index. The contributions of the low- and high-energy protons to the inner magnetosphere energy content are comparable. However, the low-energy component of the protons (<80 keV) is strongly governed by “convective” timescales (large scale convection and mesoscale, dynamic injections) and faster losses attributed to charge-exchange and flow through the magnetopause, following open drift paths and is very well correlated with the absolute value of Dst index. On the contrary, the high-energy component (>100 keV) varies on much longer timescales, initially accelerated due to adiabatic effects during the early recovery phase of the storm, and later on can be transported into the inner magnetosphere at diffusive timescales, and shows either no correlation or anticorrelation with the absolute value of Dst index, as shown in Fig. 10.

Keika et al. (2018), examined in detail the energy spectral evolution of protons (20 – 600 keV), and oxygen (> 150 keV) during the main phase of the 17 March 2015 geomagnetic storm and found that the ring current ion population contributing energies changed as the storm developed. The ring current ion population evolved in three steps: i) during the first step, an increase in the ring current energy density was predominantly contributed by 20 – 80 keV ions at L ∼ 3.5, which are due to the penetration of the pre-existing, relatively cold plasma sheet population; ii) during the second step the ring current buildup was caused due to continuing penetration of the cold plasma sheet population down to L ∼ 2.5; iii) finally, the contributing energies broadened during the third step (near the storm maximum), when energetic protons with energies greater than 100 keV at L ∼ 3 made a significant contribution. The authors attributed the third step, where the storm was intensified to a Dst level of <−200 nT, to the penetration of a hot, dense plasma sheet population generated during the course of impulsive magnetotail dynamics, similar to what was discussed in the earlier paragraphs of this section. This high-energy population (>100 keV at L ∼ 3) can survive in the deep inner magnetosphere longer than lower-energy population (<a few tens of keV) because the charge-exchange cross section for collisions with Earth’s geocorona (hydrogen neutral) is smaller by more than an order of magnitude [e.g., Ebihara and Ejiri 2003; Gerrard et al. 2014a,b]. The high-energy population may be able to continue to dominate the plasma pressure of the inner magnetosphere on a longer time scale than that of a magnetic storm, as statistically demonstrated by Gkioulidou et al. (2016). Zhao et al. (2015), who examined the contributions of the different energies and species to the total ring current energy of a moderate geomagnetic storm on March 29 2013 (minimum $\text{Dst} = -61\text{ nT}$), also arrived to similar conclusions with the above studies. They found that ions with less than 50 keV dominated the ring current during the storm main phase, while higher energy protons enhancement occurs at a later time and they dominate during the recovery phase. These findings are confirming results from previous modeling studies [e.g. Chen et al. 1994].

Singly charged ionospheric oxygen ions, can be energized up to a few tens to a few hundreds of keV, and thus significantly contributing to the plasma pressure during geomagnetically active periods. Keika et al. (2013) and references therein show pre-Van Allen Probes understanding of the possible scenarios leading to the O+ pressure abundance in the storm-time ring current. The new measurements have shed light to which processes are most important.

**Fig. 11:**
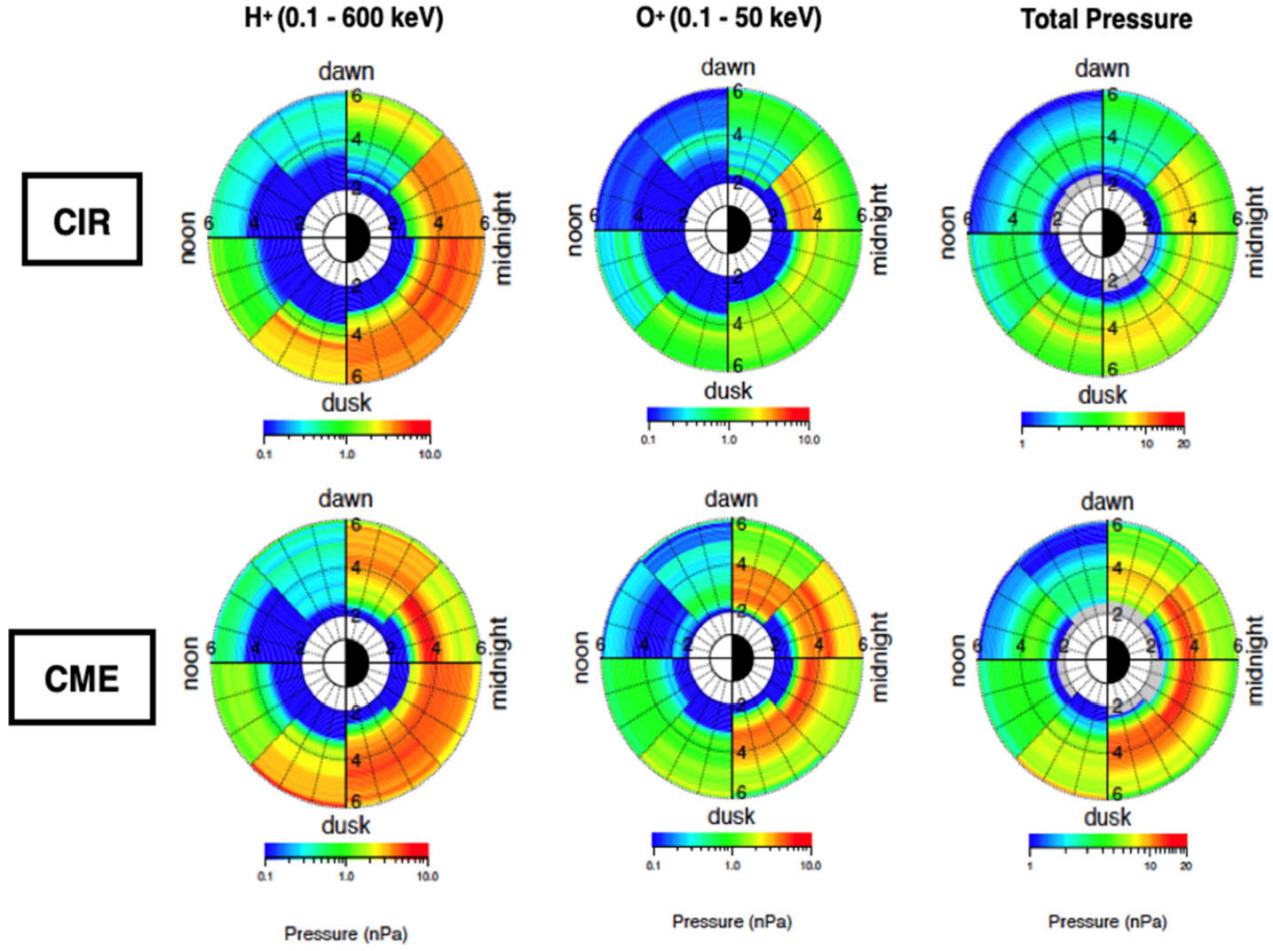
The average ring current pressure during the main phases of CIR- (top panels) and ICME- (bottom panels) driven storms; Left column: average pressure contributed by the 0.1 – 600 keV protons; Middle column: average pressure contributed by the 0.1 – 50 keV O^+^; Right column: total ion pressure (adapted from Mouikis et al. 2019)

Mouikis et al. (2019) investigated the dependence of pressure contribution of the three major ion species, protons, oxygen and helium to the storm-time ring current on solar wind drivers. They examined 25 Interplanetary Coronal Mass Ejections (ICME) and 35 Corotating Interaction Regions (CIR) moderate to intense storms (minimum Dst between −49 and −130 nT). Consistent with the studies mentioned above, they found that during the storm main phase, the major contributor to the ring current pressure in the inner magnetosphere are ions drifting duskward on open drift paths, leading to a strong partial ring current. They also found that the total ring current pressure is larger during ICME storms, and that difference in the response to solar wind drivers is attributed to the larger contribution of <∼55 keV oxygen during the main and early-recovery phases of the ICME storms (Fig. 11). The ICME oxygen pressure increases more strongly than the proton one with decreasing L and peaks at lower L shells. Menz et al. (2016) showed that the increased O+/H+ pressure ratio at low L-shells can be a result of either different spectral slope of the oxygen ions compared to protons leading to an energy dependent ratio, or the time dependence in the oxygen source. Yue et al. (2019a,b) showed that the O+/H+ pressure ratio increases during storm main phase and then rapidly decays during storm recovery phase, suggesting that O+ ion buildup and decay rates are much faster than H+ ions. They also concluded that the faster buildup of O+ ions is probably related to some species-dependent source and/or energization processes in the inner magnetosphere. On the other hand, the faster decay of O+ ions may be caused by the stronger charge exchange and Coulomb collision rates.

Keika et al. (2016) examined an injection event during the main phase of the 6 June 2013 storm, using simultaneous observations in the inner magnetosphere (L ∼ 6) at 22–23 h MLT made by two Van Allen Probes with a separation of only ∼0.5 Re. They focused on the evolution of proton and oxygen energy spectra during the event. The oxygen phase space density increased in a wide range of the first adiabatic invariant, while the spectral slope showed no significant change. Oxygen ions with energies of 0.1–10 keV measured by the Van Allen Probes HOPE instrument were enhanced prior to the storm mostly in magnetic field-aligned directions. The most reasonable scenario of this event is that warm oxygen ions supplied into the near-Earth magnetotail or the outer part of the inner magnetosphere (around GEO) prior to the storm were adiabatically transported by spatially localized, temporarily impulsive electric fields. In a similar vein, Menz et al. (2019a) and Menz et al. (2019b) investigated the effects of different electric-field models in predicting the energy dependence of the inward convection of the plasma sheet particles, during the March 17 2013 and March 17 2015 storms. They found the commonly used empirical models to be too weak in the inner magnetosphere to transport ions in the L shells they were observed, and that including the composition changes associated with substorm injections is important in capturing the pressure and composition changes in the storm-time ring current. The dynamic source combined with inward adiabatic drift explained the radial dependence of the oxygen ring current during the storm.

Even though the contribution of high energy (>100 keV) oxygen ions to the overall ring current pressure is not significant, their study can reveal processes responsible for oxygen energization in the inner magnetosphere. Mitani et al. (2018, 2019) addressed the effects of ULF waves on such energization. Mitani et al. (2018) reported that high-energy oxygen ion fluxes increased at L ∼ 3.5–4.7 during the late main phase of the 24 April 2013 magnetic storm, even though same energy proton fluxes did not. The selective oxygen increase (SOI) event was accompanied by the enhancement of poloidal ULF waves with a low $m$ number ($m< 10$, derived from magnetic field measurements on the ground at >60 ° MLAT) and frequencies of ∼1 – ∼10 mHz. Mitani et al. (2019) extended this study to include all 90 geomagnetic storms that occurred in 2013 to 2017. They defined an SOI event as an increase in oxygen phase space density (PSD) and a decrease or no change in proton PSD during a Van Allen Probes orbital period (∼9 hour). SOI events were identified in 33% of the 90 storms studied, with occurrence rate being higher during large magnetic storms ($\text{Dst minimum of} \leq -90\text{ nT}$) and with ULF wave power in the Pc4 and Pc5 frequency ranges being clearly enhanced during all of events. The enhanced ULF waves were observed at all MLT sectors inside the Alfvén layer of >100 keV ions. A likely scenario that can explain the SOI event characteristics from this statistical study is selective transport of oxygen ions due to a combination of drift-bounce resonance with Pc-4 waves at L > ∼4.5 and drift resonance with Pc-5 waves at L < ∼5.

**Fig. 12:**
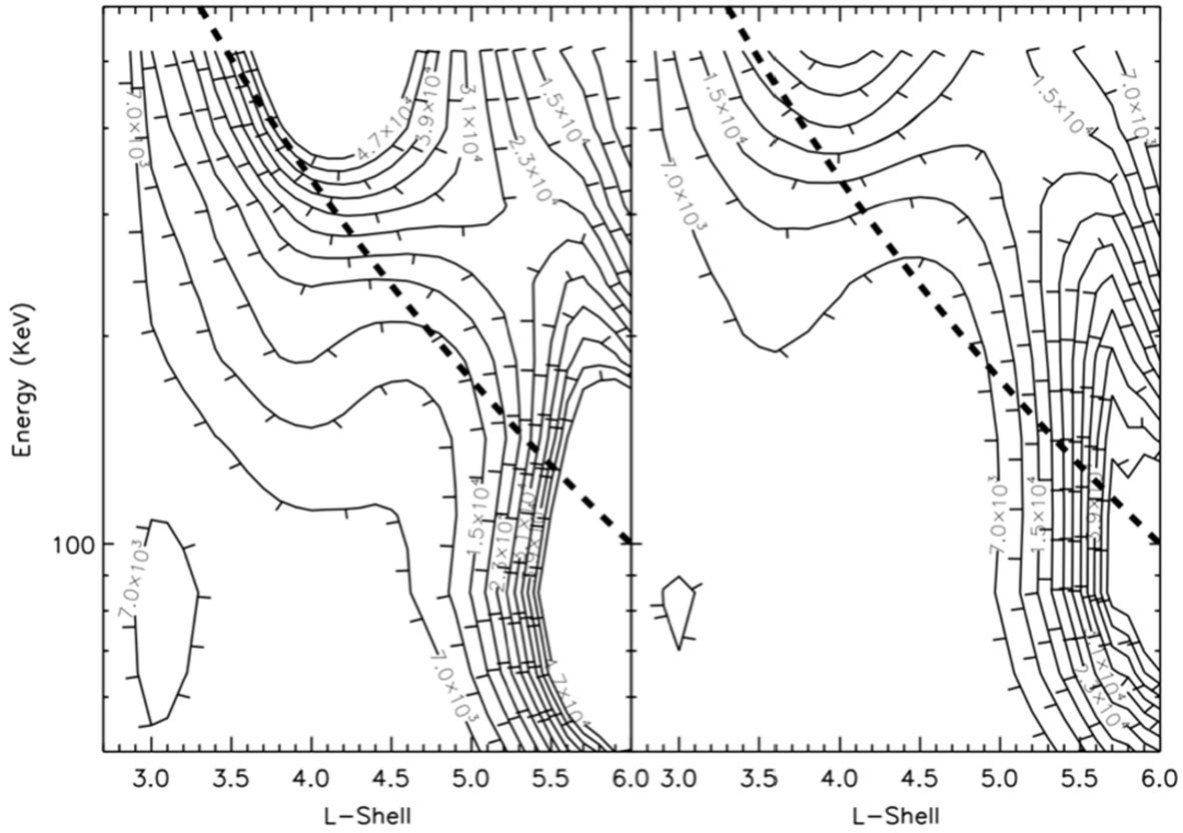
Two, sample, quiet time averages of He ion flux (particles/(s ⋅ cm^2^ ⋅ keV)) displayed in an energy/L-shell distribution. L here is determined by a dipole L model. Dashed black lines represent the first adiabatic energy trace for a 100 keV particle located at L = 6 (from Gerrard et al. 2014a)

Finally, two studies by Gerrard et al. (2014a,b) investigate the long term ion composition evolution in the inner magnetosphere. Gerrard et al. (2014a) examined the flux, L-shell, and energy (65 keV to 518 keV) morphology of ring current Helium (He) ions between geomagnetic storm injection events. They found that the overall He ion abundance during the first nine months of RBSPICE measurements, the appearance of a persistent high energy, low L-shell He ion population, and the temporal evolution of this population all provide new insights into trapped ring current energy He ions (see Fig. 12). Of considerable note was the discovery of two distinct He ion belts, a hot inner torus and a colder outer torus. These data provide a unique resource that are important to provide verifications of, and improvements to, models of He ion transport and loss in Earth’s ring current region. In a follow up study, Gerrard et al. (2014b), investigated protons (∼45 keV to ∼600 keV), He ions (∼65 keV to ∼520 keV), and Oxygen (O) ions (∼140 keV to ∼1130 keV) integral flux measurements from the RBSPICE instrument, and form a cohesive picture of ring current ions during the first nine months of the Van Allen Probes mission. The data show injection characteristics via the He-ion/H-ion abundance ratio and the O-ion/H-ion abundance ratio. Of unique interest to ring current dynamics are the spatial-temporal decay characteristics of the two injected populations. The He-ions decay more quickly at lower L shells, on the order of ∼0.8 day at L shells of 3–4, and decay more slowly with higher L shell, on the order of ∼1.7 days at L shells of 5–6. Conversely, O-ions decay very rapidly (∼1.5 h) across all L shells. The He-ion decay time are consistent with previously measured and calculated lifetimes associated with charge exchange. The O-ion decay time is much faster than predicted and is attributed to the inclusion of higher-energy (> 500 keV) O-ions in the decay rate estimation. These measurements demonstrate a compelling need for calculation of high-energy O-ion loss rates, which have not been adequately studied to date.

### Properties of Energetic Ion Injections in the Inner Magnetosphere and Their Physical Implications

As it has been discussed in the section above, energetic ion injections inside geosynchronous orbit can be ubiquitous throughout the main phase of geomagnetic storms. Therefore, understanding the formation and evolution of energetic particle injections is critical in order to quantify their effect in the inner magnetosphere. Several studies incorporating Van Allen Probes RBSPICE and HOPE data as well as simulations, have investigated the properties of those injections and the physical processes that shape those properties.

Gkioulidou et al. (2015) presented a study of two distinct energetic particle injections, being observed inside geosynchronous orbit during a weak storm, occurring 10 min apart, yet exhibiting different dipolarization signatures and durations. For their study they combined multipoint in-situ particle and magnetic field observations from inside geosynchronous orbit to the plasma sheet, as well as ground magnetometer data. They found that the first injection, a dispersionless, short-timescale (∼ 3 min), energetic proton and electron injection, accompanied by a sharp dipolarization of the magnetic field, is associated with a weak current system possibly caused by a localized low-entropy bubble extending ∼ 2 h in MLT. In fact, the in-situ measurements of that injection exhibit all the characteristics of a dipolarization front, typically observed in the near-Earth plasma sheet, penetrating deep inside geosynchronous orbit: sharp dipolarization of the magnetic field, increase of the pressure and density right ahead of the front, decrease of the pressure and density and increase of temperature right behind the front, and sharp decrease of the entropy to lower than pre-dipolarization values. On the contrary, the second injection, a dispersed in energy proton injection, lasting ∼ 10 min, accompanied by a gradual dipolarization, is found to be associated with the development of a large-scale substorm current wedge. The second injection also exhibits similar changes in the plasma properties before and after the dipolarization, but over longer timescales. Turner et al. (2017) also followed a multi-point observations approach in order to investigate the morphology and evolution of energetic particle injections associated with substorm activity. Observations of ion and/or electron dispersed injections from 16 satellites, including the two Van Allen Probes, spread both in MLT and radial distance, were combined with drift mapping analysis using a simplified magnetic field model, in order to get an estimate of when and where the initial, dispersionless injection boundary must have occurred. Their analysis revealed that multiple, localized in MLT electron injections, preceded, and penetrated deeper (inside geosynchronous orbit) than, a more global injection of both ions and electrons. However, the authors also noted discrepancies in the number, penetration depth, and complexity of electron compared to the ion injections. Both Gkioulidou et al. (2015) and Turner et al. (2017) studies revealed how multipoint in-situ observations are both necessary but, at the same time, probably insufficient in order to understand the nature and the variable spatial and temporal scales of energetic particle injections as they emerge and evolve throughout the magnetosphere.

**Fig. 13:**
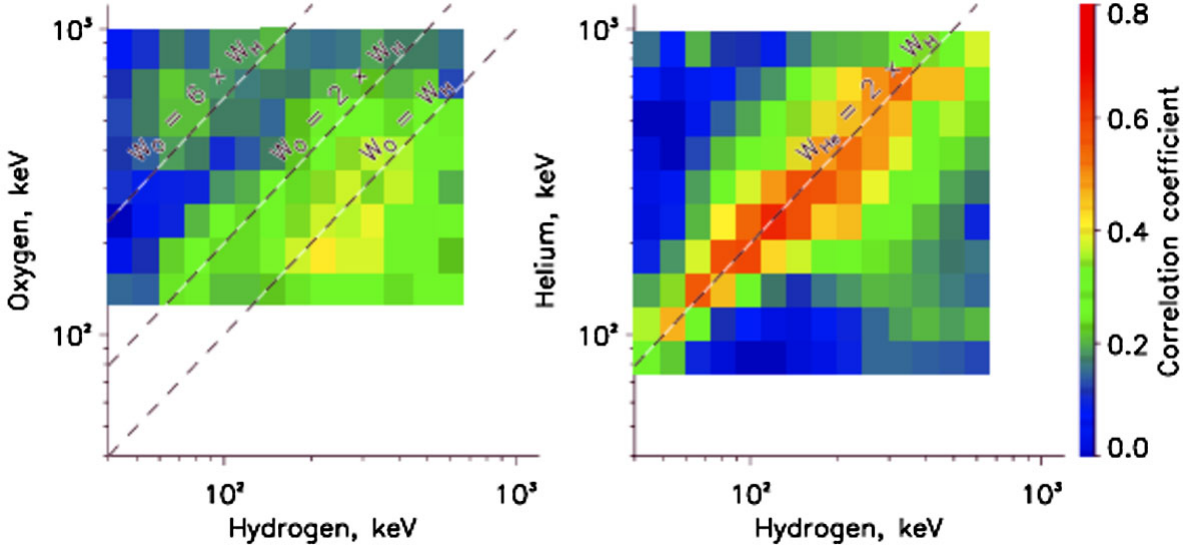
Average correlation coefficient patterns of (left) the oxygen and hydrogen (O-H) ion flux variations and (right) the helium and hydrogen (He-H) ion flux variations at the RBSPICE energy channels (>50 keV). For reference, dashed lines are denoted at the common O-H energy channels (E$_{\mathrm{O}} = E_{\mathrm{H}}$) and at the energy channels of which the O energy is twice the H one (E$_{\mathrm{O}} = 2 x E_{\mathrm{H}}$) and 6 times the H one (E$_{\mathrm{O}} = 6 x E_{\mathrm{H}}$) for the O-H pattern and at the energy channels of which the He energy is twice the H one (E_He_ = 2 x E_H_) for the He-H pattern (from Motoba et al. 2018)

**Fig. 14:**
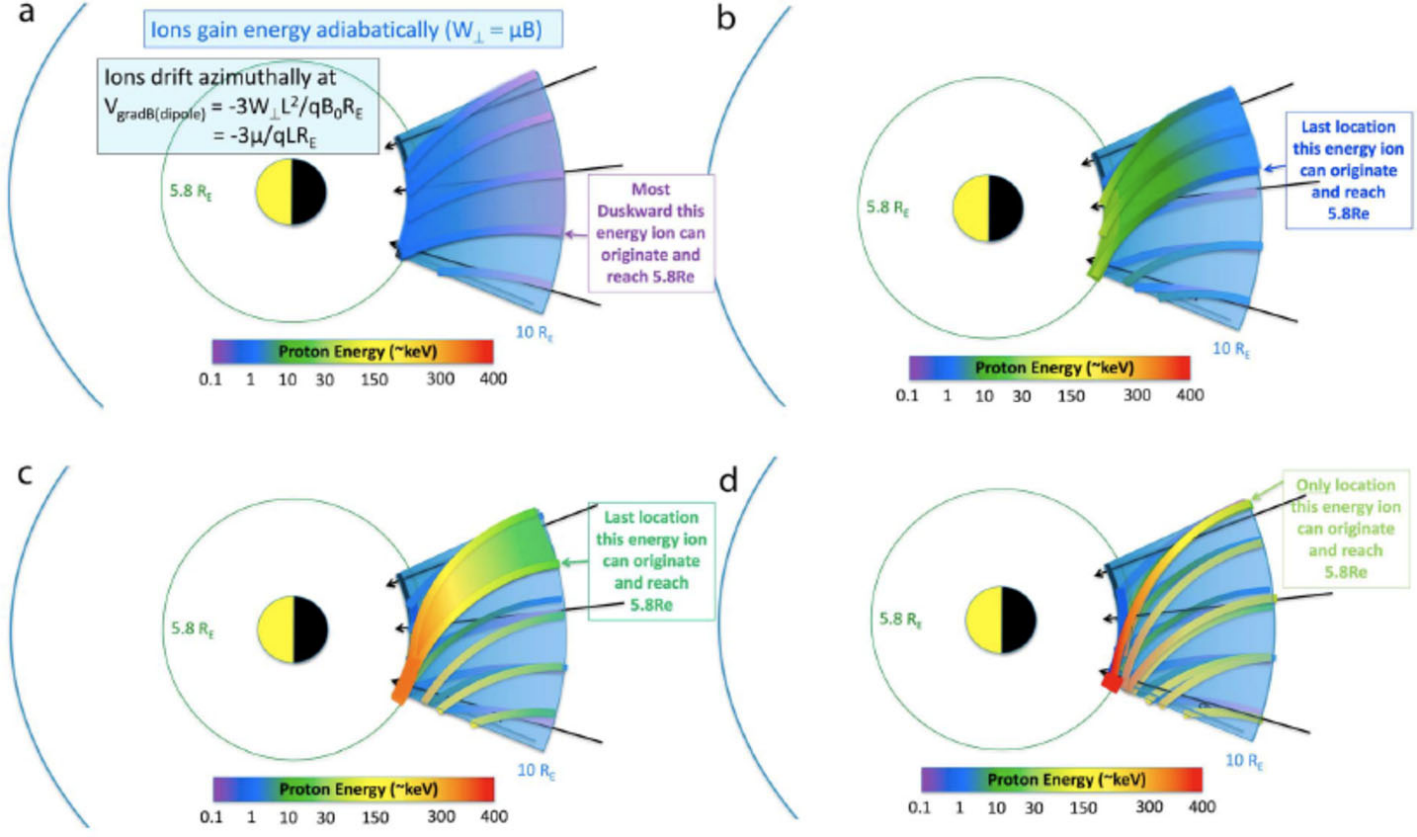
Cartoon model ion trajectories depicting the convection- and drift-dominated, charge-dependent adiabatic energization as discussed in Mitchell et al. (2018). Ions gain energy adiabatically as they are convected Earthward. They drift azimuthally in the radial gradient of the magnetic field. Their energy gain can also be calculated by the expression q × E × d, where q is the ion charge state, E is the azimuthal electric field in the channel, and d is the azimuthal distance through which they have gradient drifted during their transport (from Mitchell et al. 2013)

A multi-case study of energetic ion injections by Mitchell et al. (2018), showed that the energization, peak energy, and drift dynamics are well ordered in energy/charge, that is, O^+^ gains the same amount of energy as H^+^, He^++^ gains twice that energy, and O^6+^ gains 6 times that energy. More specifically, most of the helium injected is doubly ionized (He^++^), while oxygen can be other singly (O^+^) or six times (O^+6^) ionized, indicating the existence of both ionospheric and solar wind (at a smaller abundance) origin populations. These findings were also confirmed by Motoba et al. (2018) statistical study of energetic injections associated with dipolarizations inside geosynchronous orbit as it is shown in Fig. 13. Mitchell et al. (2018) concluded that the observed injection phenomenology can be explained by nearly adiabatic transport within an azimuthally localized flow channel, where gradient/curvature drift causes the highest energy particles to exit the channel before the injection reaches the Van Allen Probes location, thus it limits the highest energy/charge observed for each injection. In fact, even at the highest measured ion energies where gyroradius and scattering effects might be expected to appear, energization depends on charge state but not on ion mass. Figure 14 shows a cartoon model of ion trajectories demonstrating how a simple adiabatic model can lead to the charge-dependent energization of the observed injections. The different panels represent different initial energies at the 10 R_E_ boundary, i.e. a) 0.1 keV, b) 2-3 keV, c) 20 keV, d) the very highest energies that can be transported from the outer boundary (10 R_E_) to the inner boundary (5.8 R_E_, i.e. Van Allen probes apogee). Due to stronger magnetic drift at higher energies, the highest the energy at the outer boundary, the narrower local time segment of the flow channel can reach the inner boundary.

**Fig. 15:**
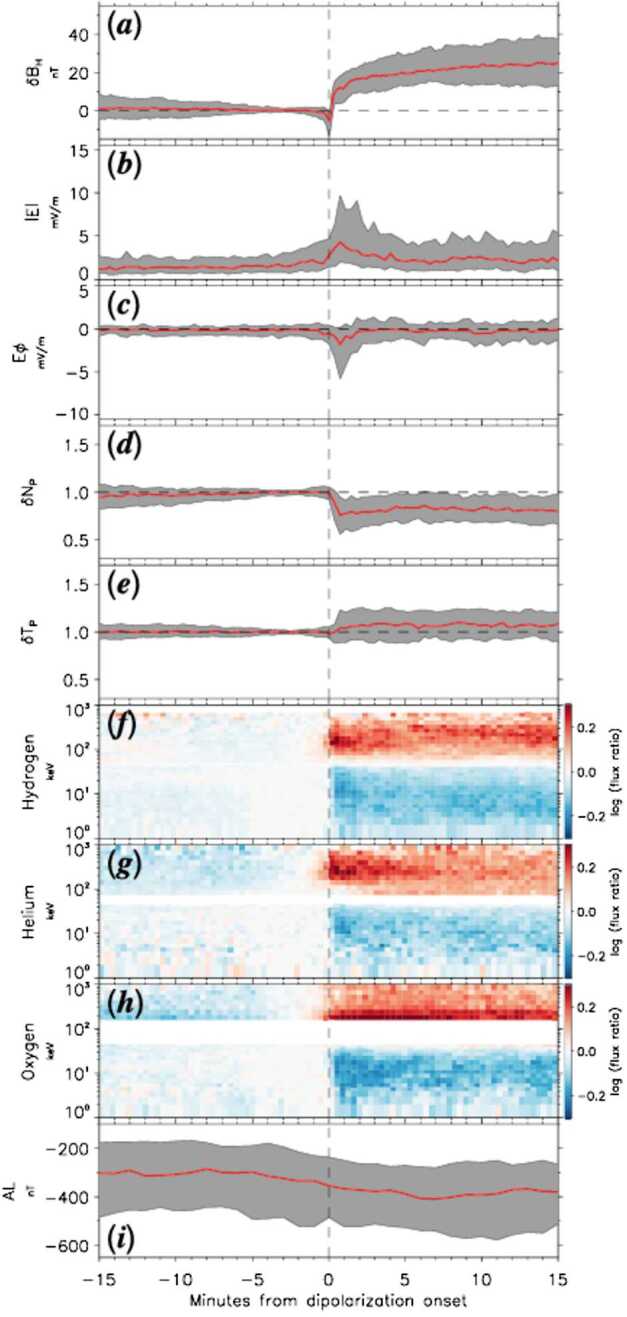
Superposed epoch results of Van Allen Probes measurements for the 144 dipolarization events. From top to bottom, (a) B_H_ variations ($\delta $B_H_), (b) total electric field strength (|E|), (c) the azimuthal component of electric field (E$\varphi $, positive eastward), (d) normalized proton number density ($\delta \text{ N}_{\mathrm{P}}$), and (e) temperature ($\delta $T_P_) variations, (f) hydrogen, (g) helium, and (h) oxygen ion flux ratios around 90° pitch angle to the corresponding pre-onset levels, and (i) AL index. The red trace and gray shading area in Fig. 15a–15e and 15i are the median value and interquartile range of these parameters, respectively. The red (blue) color code in Fig. 15f–15h represents the relative flux increase (decrease) to the pre-onset level, given on a logarithmic scale (from Motoba et al. 2018)

Two studies by Motoba et al. (2018, 2020) statistically investigated the relationship between dipolarizations and energetic particle injections inside geosynchronous orbit. Motoba et al. (2018) conducted a statistical epoch analysis of hydrogen, helium, and oxygen ion fluxes at 1-1000 keV around the onset of 144 dipolarizations events that occurred at magnetic inclination > 30 ° during the 2012-2016 tail seasons’ observations of the Van Allen probes. They found that several properties accompanying the magnetic dipolarization inside geosynchronous orbit, morphologically resemble those around dipolarization fronts in the near-Earth tail. That is, as shown in Fig. 15, i) they are preceded by a transient decrease of the northward magnetic field component, ii) they are accompanied by a transient impulsive westward electric field component enhancement, a decrease in the proton density and increase in the proton temperature, iii) they are accompanied by energy dependent flux increase (decrease) for energies above (below) ∼ 50 keV for all ion species (proton, helium, and oxygen). Nonetheless, there are also different responses among the different ion species to the dipolarizations. i) The ratio of the flux after, to that before the onset of the dipolarization peaks at different energies for helium (200 – 400 keV), and protons and oxygen ions (100 – 200 keV), indicating the charge dependent energization of the solar wind originating H, He^++^ and ionospheric O^+^ populations, similarly to the Mitchell et al. 2018 findings discussed above. ii) While the protons and helium flux ratios increase after the onset occurs sharply, within 2 min of the onset, and is short-lived the oxygen flux ratio reaches its peak with a few minutes delay compared to protons and helium and it takes longer to decay. iii) Closer to the earth (r < 5 R_E_) the oxygen flux ratio peak surpasses the proton and helium one. iv) The enhanced ion flux ratio peak and its energy are higher for sharp dipolarization than gradual one, suggesting the importance of the accompanying electric field in the efficient transportation and/or energizion of the ions in the inner magnetosphere. Motoba et al. (2020) using the same 144 dipolarization events, conducted a superposed epoch analysis to investigate the pitch angle dependence of the flux changes of electrons, hydrogen, helium, and oxygen, after the onset of the dipolarization.

They found that for electron energies > 80 keV the flux increases primarily around Pitch Angle (PA) = 90 °, while for energies 10 – 50 keV the flux increase is almost isotropic and for energies < 5 keV, there is field-aligned flux increase. Similarly to electrons, ions of energies > 80 keV exhibit post-onset flux increase at PA = 90 °. However, contrary to electrons, for ions of energies below < 30 keV the post-onset flux decreases independent of PA. Only low-energy, < 5 keV, helium and oxygen exhibit strong field-aligned enhancement post-onset, also similarly to electrons of similar energies, indicating possible ionospheric outflow events. The Motoba et al. (2018, 2020) studies reveal a comprehensive picture of the effect dipolarizations inside geosynchronous orbit can have on different particle species, energies and pitch angle distributions, and the implications of this morphological behavior with respect to various processes of particle transport and acceleration within the inner magnetosphere.

**Fig. 16:**
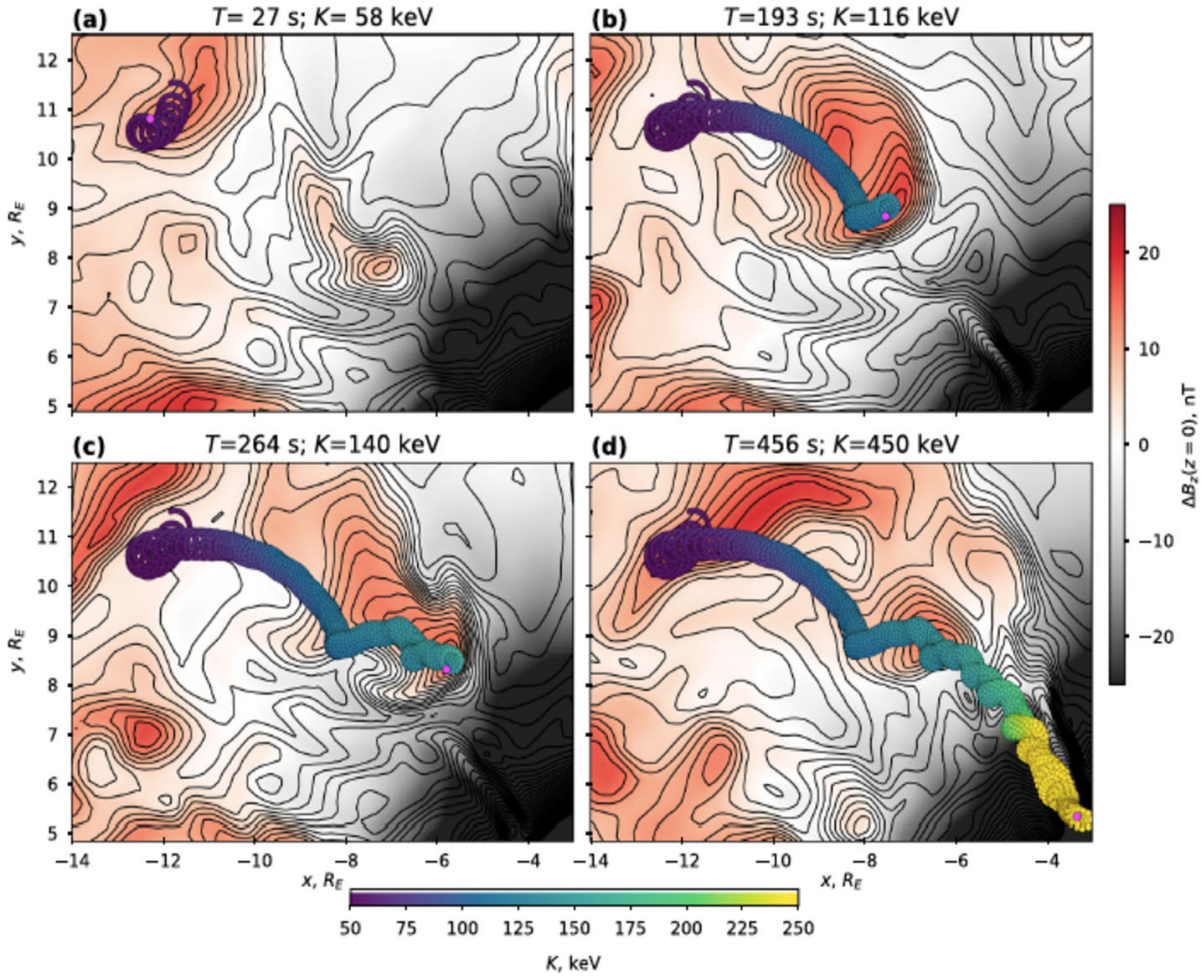
Proton trapping and acceleration at an isolated dipolarization front. Panels (a)–(d) show snapshots of the proton trajectory at different times of the simulation projected onto the equatorial plane; each snapshot shows the trajectory from T = 0 to the instance indicated by the magenta circle. Evolution of particle energy along the trajectory is indicated with color. The external magnetic field, $\Delta $B$z$, is shown with color. Contours of constant total magnetic field for each snapshots are shown with black lines (from Ukhorskiy et al. 2018)

Ukhorskiy et al. (2018), using three-dimensional test-particle simulations, traced ions within high-resolution global magnetohydrodynamic (MHD) fields in order to investigate their transport and energization within mesoscale dipolarizations from the plasma sheet to the inner magnetosphere. They showed that ions can get trapped within strong magnetic field gradients at the interface between the dipolarizations and the ambient plasma. In particular, magnetically trapped plasma sheet protons of energies above 5 – 10 keV can get transported more than 10 Earth radii inward and get accelerated by ∼ a factor of 10, reaching higher energies compared to a purely adiabatic energization process. An example of such ion trapping, transport and energization in a single dipolarization front is shown in Fig. 16. Similarly to the findings by Gkioulidou et al. (2014), and Yang et al. (2015), the magnetically trapped proton population can have significant contribution to the ring current, ranging from 20% - 60%, depending on the plasma sheet temperature and energy spectrum. The authors also investigated how the trapping affects different species, and confirmed the Mitchell et al. (2018) and Motoba et al. (2018) observational findings that the ion acceleration is proportional to the ion charge and independent of the mass.

### Wave – Ion Interactions

Interactions of energetic (>10 keV) ions with magnetospheric ULF waves have been both theoretically and observationally addressed for more than half a century. In addition to the global aspects of the interactions and their effects on inner magnetosphere dynamics, the measurements of energetic ions taken by RBSPICE yielded important insights into physical mechanisms of ULF wave-ion interactions - particularly energy transfer between waves and ions through drift-bounce resonance interactions.

Yamamoto et al. (2019), Yamamoto (2020) examined two wave packets of second harmonic poloidal Pc-4 waves (∼7 mHz) observed at $r \sim 5.8$ Re and 13 hr MLT near the magnetic equator. Energetic proton measurements by RBSPICE showed butterfly pitch angle distributions with energy dispersions. 10-30 keV proton fluxes oscillated at the same Pc-4 frequency. It was found, based on the ion sounding technique (Min et al. 2017), that the two wave packets propagated eastward with an azimuthal $m $ number of ∼220 and ∼260, respectively. The waves were excited by substorm injections of 10-30 keV protons with pitch angles of about 40/140 ° through drift-bounce resonance interactions. It was also determined that the radial gradient of proton phase space density, $\partial f$/$\partial L$ (rather than the energy gradient, $df$/$dW$) played the dominant role in generating the two wave packets. The cold electron density was >100 cm^−3^ around the Van Allen Probes apogee during the events; the high density decreases the eigenfrequency of the Pc-4 waves and thus causes an increase in the $m$ number.

Oimatsu et al. (2018) examined poloidal Pc-4 waves and associated same-frequency oscillations of energetic proton (67 - 269 keV) fluxes observed on the dayside near the magnetic equator at 1600-2000 UT on 2 March 2014. A phase difference was seen between the waves and the flux oscillations; the phase angle depends on the proton energies and pitch angles. The dependence observed in the data permitted an estimate of the resonance energy to be 170–180 keV for $\alpha = 90$ °±40 ° and ∼120 keV for $\alpha = 90$ °±60 °. The calculation of the energy gradient ($\partial f$/$\partial W$|$\mu $,$L$) and spatial gradient ($\partial f$/$\partial L$|$\mu $,$W$) of the proton phase space density gave $df$/$dW > 0$ on both the inbound and outbound legs of the Van Allen Probes orbit. Oimatsu et al. (2018) suggested that the drift-bounce resonance ($N = 1$) during this event transfers energy from the protons to the waves. The proton energy loss was estimated to be up to ∼85% of the total energy loss of the ring current, which was estimated from the increase of the Dst* index, ∼6.7 nT.

An asymmetry of high plasma beta ($\beta > 1$) occurrences in the inner magnetosphere, with preference for the dusk-to-midnight sector of the inner magnetosphere (Cohen et al. 2017), was found using calculated pressures and magnetic field data provided by RBSPICE and EMFISIS instruments respectively. Additional investigation showed there to be a statistically high probability of high-$\beta $ plasma regions in the dusk-to-midnight sectors also having sufficiently high plasma kappa ($\kappa > 0$), indicative of plasma instability to mirror and drift-mirror waves (Cooper et al. 2021). These high-beta, high-kappa plasmas are a likely important source of ULF waves in the dusk-to-midnight sector, often associated with “storm-time ULF waves.”

**Fig. 17:**
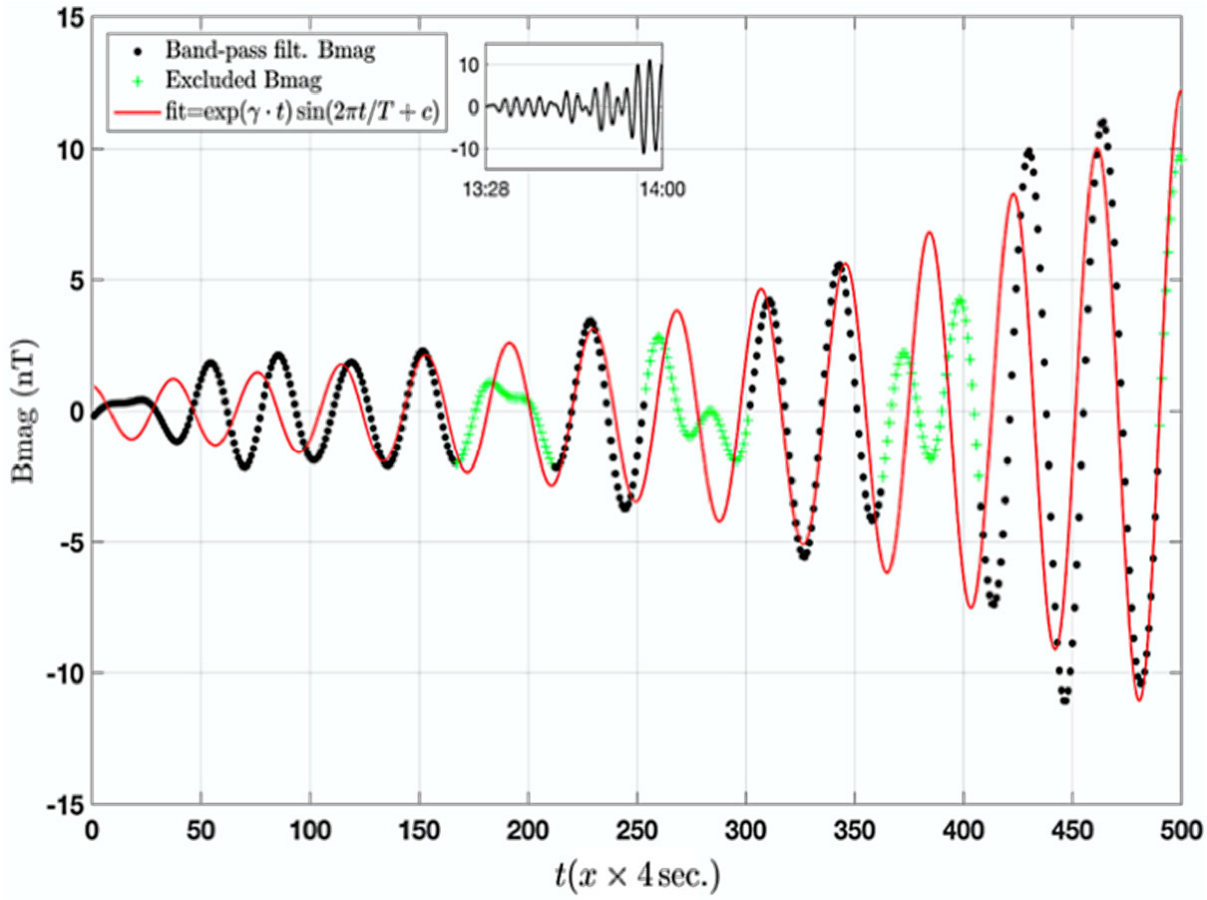
Top inset: The band-pass filtered (3 dB at 4 mHz and 10 mHz cutoff frequencies) total magnetic field (from 13:28 – 14:01 UTC). Magnified magnetic field data (black dots), same as the inset, obtained by the 4 s. resolution EMFISIS-B instrument. The red line is the fitted function using the Least Absolute Residual (LAR) robust nonlinear squares method, and assuming predicted growth rate, $\gamma $. The green crosses represent the outliers. The x-label represents the time elapsed since 13:28 UTC. The goodness of fit gives an r-square of 0.79, meaning that the parameters ($\gamma $, $T$, $c$) obtained can explain about 79% of the variance in the measured wave data (excluding the outliers, here identified as higher frequency components) (from Soto-Chavez et al. 2019)

Soto-Chavez et al. (2019), reported on the strong evidence for the generation of a ULF wave by the drift-mirror plasma instability in the dynamic plasma environment of Earth’s inner magnetosphere. Theoretical analysis of the plasma and wave observations demonstrated that the drift-mirror mode plasma instability condition is well satisfied, and that the measured wave growth rate agrees well with the predicted linear theory growth rate, as shown in Fig. 17. Hence, the in-situ space plasma observations and theoretical analysis revealed that local generation of ULF and high amplitude plasma waves can occur in the high beta plasma conditions of Earth’s inner magnetosphere.

**Fig. 18:**
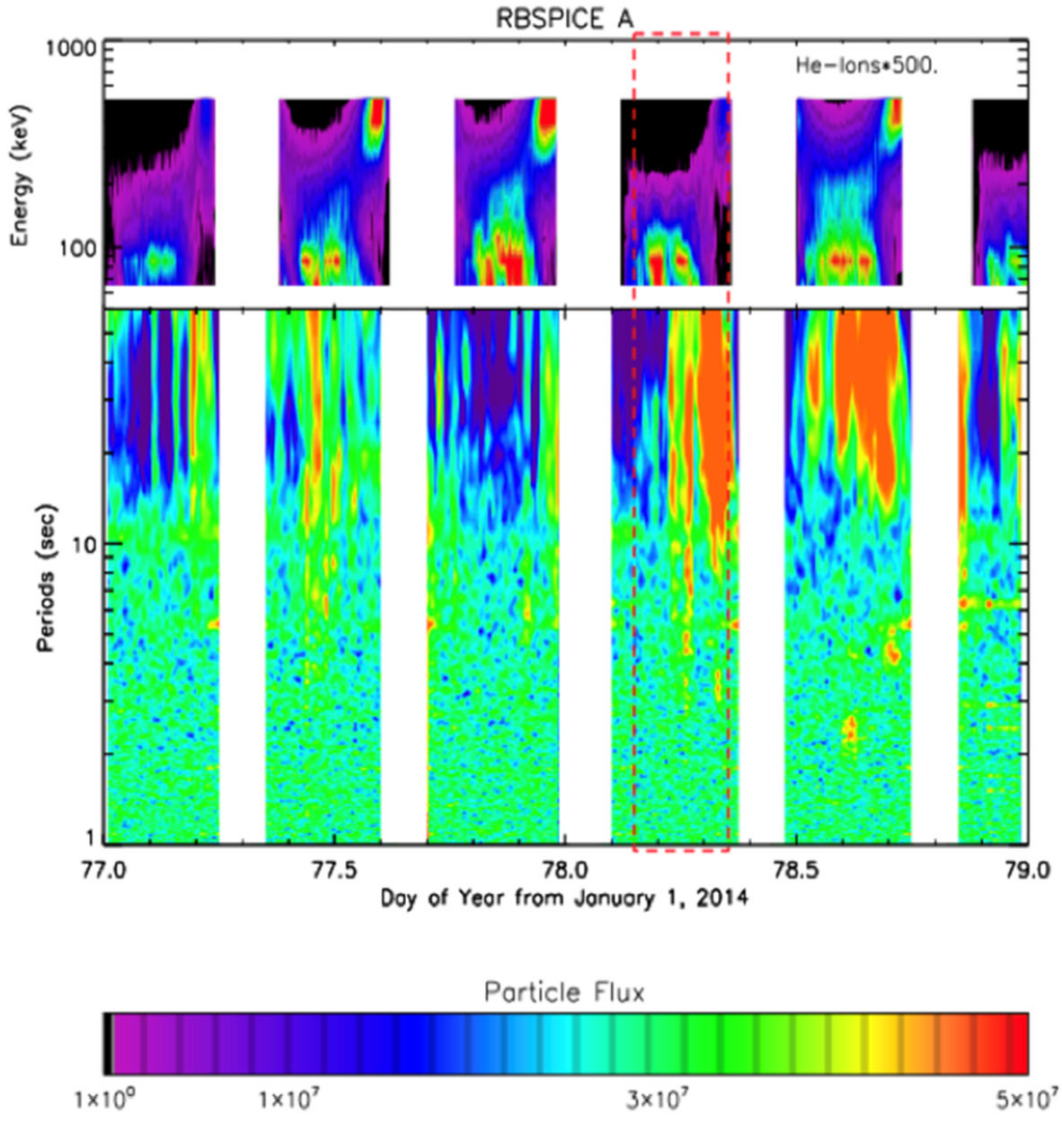
(top) The 90-deg pitch angle He ion differential flux measurements. (bottom) Parallel magnetic field component power spectrograms from the EMFISIS instrument aboard Spacecraft A. Flux values in He ions have been multiplied by a factor of 500 for use of a common color scale. The relative magnetic field power spectrogram ranges on a log scale from 0.1 (black) to 5.0 (orange) in value (from Kim et al. 2017)

Kim et al. (2017) reported considerable variability of 90 pitch angle He ion flux in the energy range of 85-142 keV during quiet times. Using data from one full spacecraft precession (2013 to 2014) they showed that the flux variation is anticorrelated with the compressional component of ULF wave power as shown in Fig. 18. The study suggests the bounce resonant pitch angle scattering mechanism is a major component for the scattering of He ions during quiet times. This is relevant information about baseline mechanisms governing ring current particle dynamics in Earth’s magnetosphere and the He ion population in the ionosphere.

**Fig. 19:**
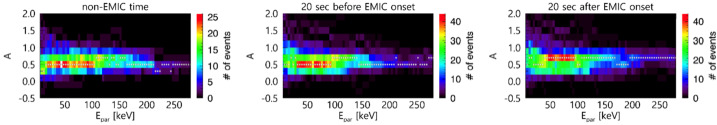
Distributions of the Kennel-Petschek (KP) anisotropy A as a function of the parallel kinetic energy E_par_. From left to right, the results are shown for non-EMIC wave times and the times 20 seconds before and after EMIC wave onsets, respectively. The white dots indicate the most likely anisotropy values at each parallel energy. The bin size for the kinetic energy is 4 keV and that for the KP anisotropy is 0.2 (from Noh et al. 2018)

Noh et al. (2018) investigated the proton anisotropy parameter for EMIC waves, a theoretical concept originally developed by Kennel and Petschek (1966). Unlike the temperature anisotropy, it is found that the energy-dependent anisotropy changes during the wave onset using the original formula. Using data from the EMFISIS, RBSPICE and HOPE instruments on the Van Allen Probes spacecraft, a statistical survey was conducted to compare the anisotropy parameter 20 seconds after the wave onset with measurements 20 seconds before the onset and during an interval with no EMIC waves (Fig. 19). The survey shows a statistically small (∼10%) increase of the anisotropy in a certain parallel energy range (∼10 – 100 keV) while a large fraction of EMIC waves satisfies the ion cyclotron instability criterion. The results indicate that the plasma state is usually near the marginal state of the ion cyclotron instability.

## Concluding Remarks

The first part of this paper discusses in-flight calibrations and lessons learned from the RBSPICE instrument’s seven years of operations, therefore it is very complementary to the Mitchell et al. (2013) manuscript, which described in great detail the instrument itself, and calibrations performed in the lab. The second part of this paper, focuses on the scientific advances enabled by the Van Allen Probes composition data. The Van Allen Probes Mission provided a transformational view of Earth’s space environment during its seven-year operation. The RBSPICE instrument as an essential and integral part of the Van Allen Probes comprehensive, unique instrumentation, provided critical data that shed light to ring current dynamics and composition. In particular, the unprecedented temporal resolution, in combination with the continuous sampling of the inner magnetosphere within geosynchronous orbit, led to new discoveries about the nature of dynamic, energetic ion injections in the inner magnetosphere, as well as their physical implications, such as their potential contribution to the total energy content in the near-Earth space environment during geomagnetic storms, or their ability to generate waves that subsequently shape the radiation belts.
